# Supplementary material for: Are cross-sectional safety climate survey results in operating room staff associated with the surgical site infection rates in Swiss hospitals?
Source: BMJ Open. 2023 Apr 19;13(4):e066514. doi: 10.1136/bmjopen-2022-066514 (PMC10124250; doi:10.1136/bmjopen-2022-066514)
Supplement: Supplementary data [file bmjopen-2022-066514supp001.pdf]

Supplementary Material

Figure S1: Patient cascade and selection for hip and knee , colorectal and cesarean section procedures for the safety climate endpoint (complete case analysis).

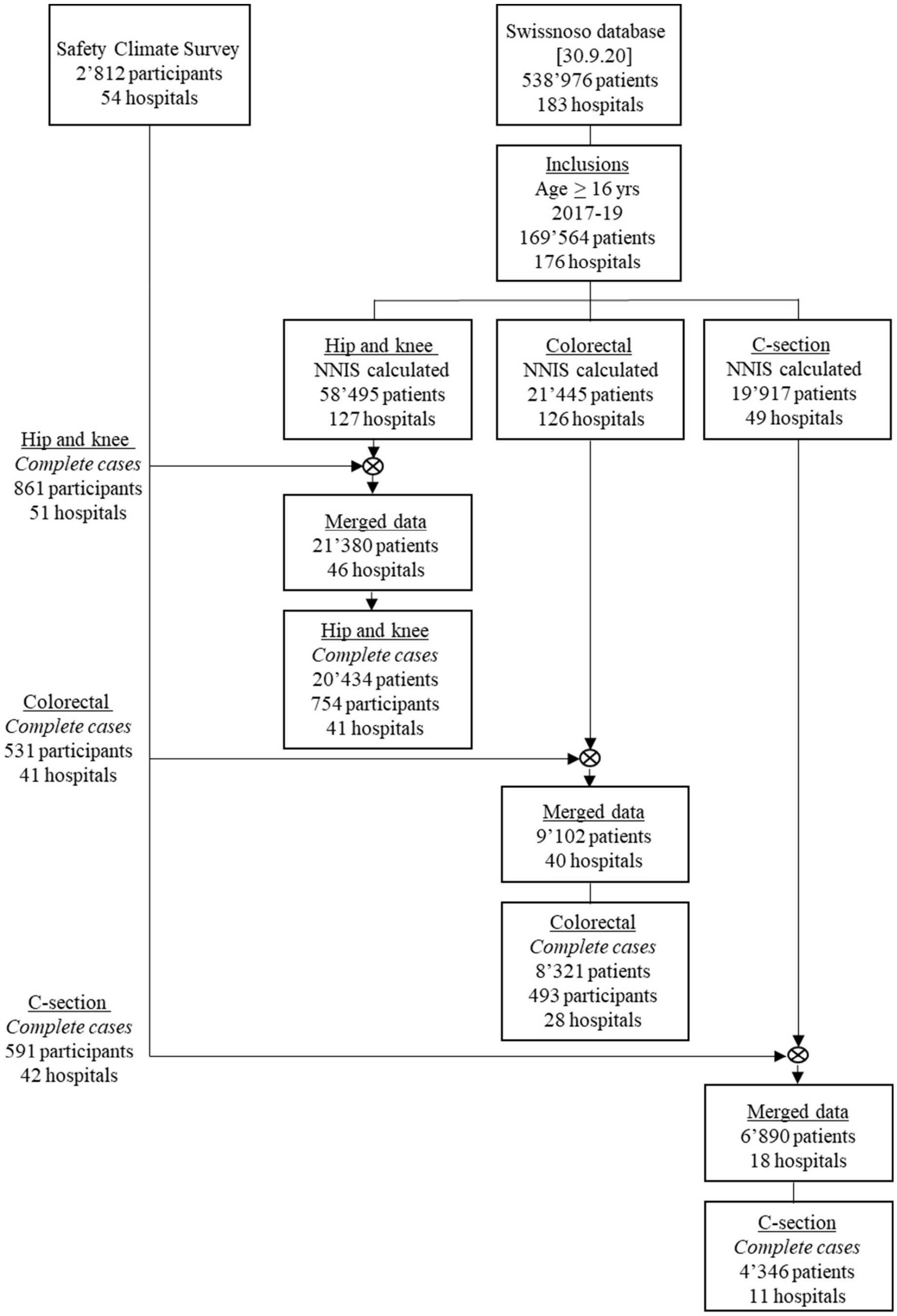

Figure S2A: Boxplot of calculated NNIS adjusted SSI rates per hospital, stratified by surgery type

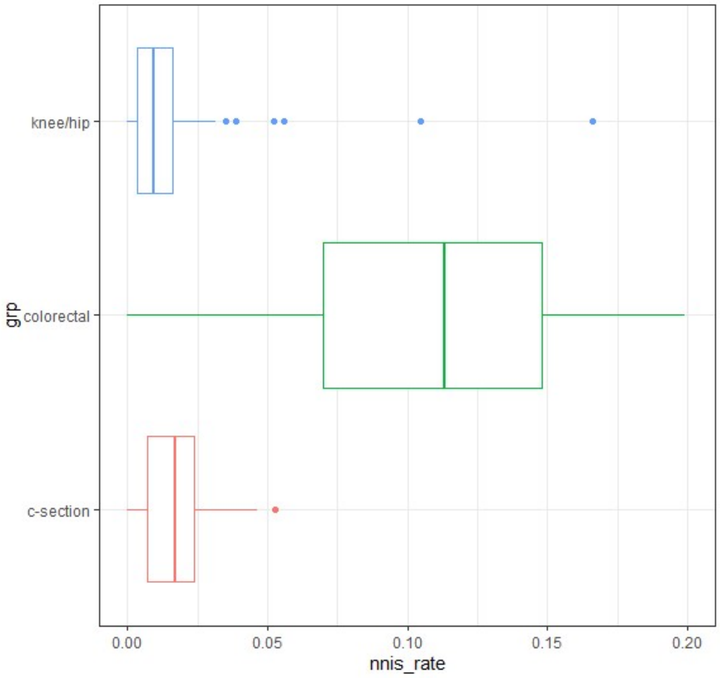

Pfeiffer Y, *et al. BMJ Open* 2023; 13:e066514. doi: 10.1136/bmjopen-2022-066514

Figure S2B: Boxplots of calculated percentage safety climate (left) and teamwork (right) per hospital, stratified by surgery type for the complete cases from the participants

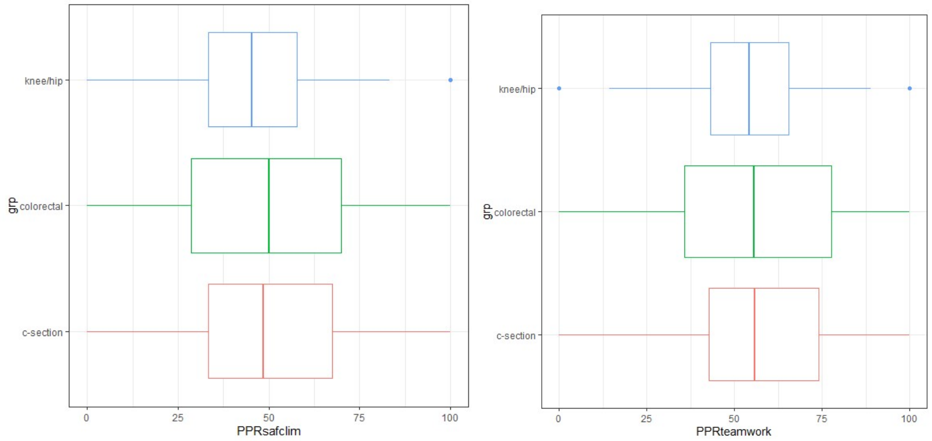

Pfeiffer Y, *et al. BMJ Open* 2023; 13:e066514. doi: 10.1136/bmjopen-2022-066514

Figure S3: Safety climate (A) and teamwork (B), stratified by hospital size

Hip and knee arthroplasty

A: Safety climate and hospital size

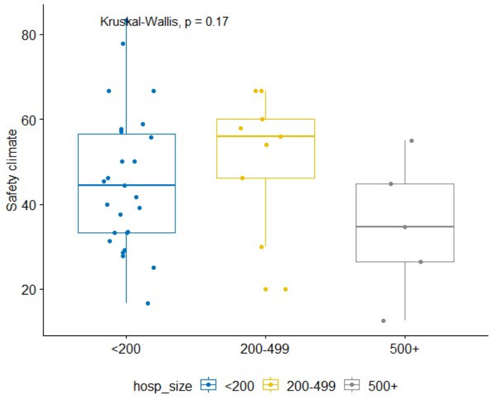

B. Teamwork and hospital size

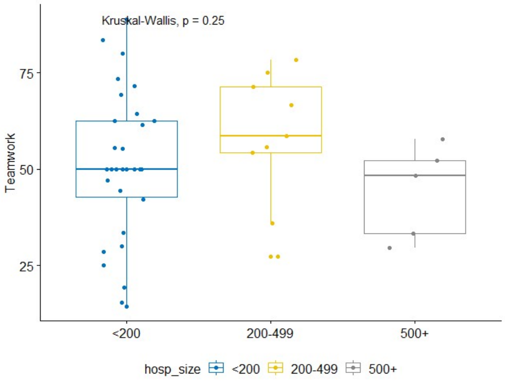

C. Infection and hospital size

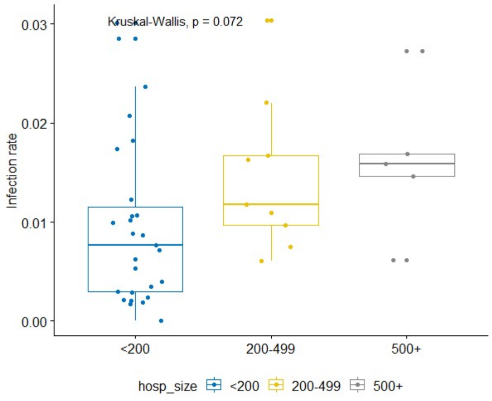

<u>Colorectal</u>

A: Safety climate and hospital size

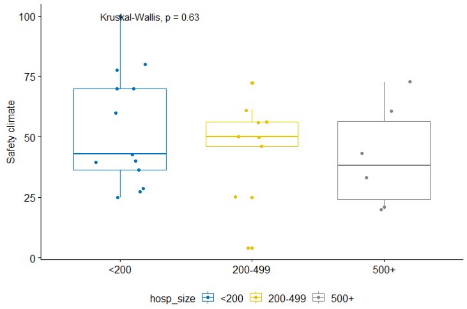

B. Teamwork and hospital size

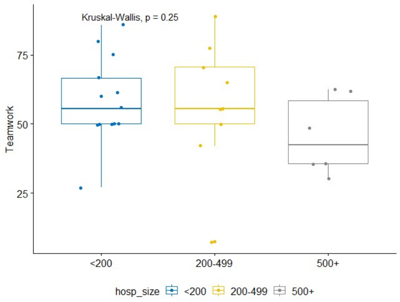

C. Infection and hospital size

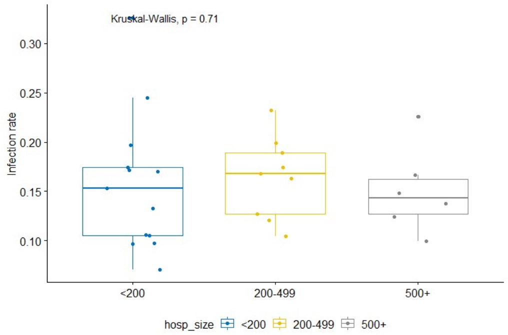

Cesarean section

A: Safety climate and hospital size

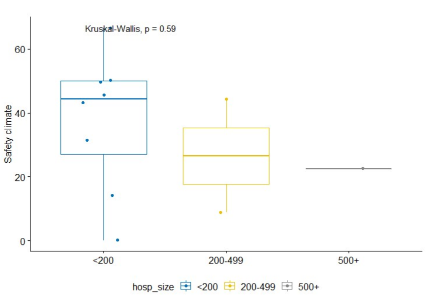

B. Teamwork and hospital size

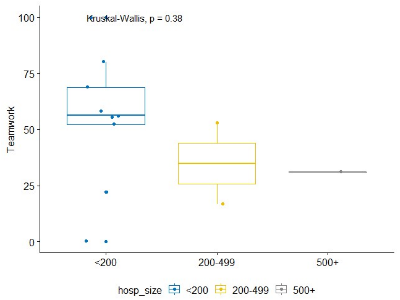

C. Infection and hospital size

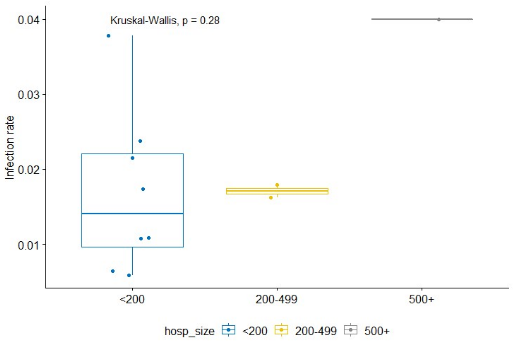

Pfeiffer Y, *et al. BMJ Open* 2023; 13:e066514. doi: 10.1136/bmjopen-2022-066514
