# Supplementary material for: Are cross-sectional safety climate survey results in operating room staff associated with the surgical site infection rates in Swiss hospitals?
Source: BMJ Open. 2023 Apr 19;13(4):e066514. doi: 10.1136/bmjopen-2022-066514 (PMC10124250; doi:10.1136/bmjopen-2022-066514)
Supplement: Supplementary data [file bmjopen-2022-066514supp003.pdf]

Supplementary Tables

Table S1: Patient characteristics for all hip and knee arthroplasties, colorectal, and Cesarean section procedures in 2017-19

**Characteristic**<br>N (%) / median [IQR]	**Hip and knee**	**Colorectal**	**Cesarean section**
Number of surgeries	58’495	21’445	19’917
Number of hospitals	127	126	49
Surgical site infection	564 (1.0%)	2’973 (13.9%)	405 (2.0%)
Hospital size (beds)			
<200	42’200 (72.1%)	8’625 (40.2%)	13’991 (70.2%)
200-499	13’640 (23.3%)	8’494 (39.6%)	5’137 (25.8%)
500+	2’655 (4.5%)	4’326 (20.2%)	789 (4.0%)
Procedure Type			
Knee	24’711 (42.2%)		
Rectal		888 (4.1%)	
Class			
I (clean)	58’495 (100.0%)	0 (0.0%)	0 (0.0%)
II (potentially contaminated)	-	14’835 (69.2%)	14’281 (71.7%)
III (contaminated)	-	2’548 (11.9%)	5’235 (26.3%)
IV (infected)	-	4’062 (18.9%)	401 (2.0%)
Duration of procedure (mins)	80 [63, 102]	165 [120, 223]	40 [31, 51]
Age (years)	70 [61, 76]	69 [58, 77]	33 [30, 37]
Sex – female	32’226 (55.1%)	10’849 (50.6%)	19’917 (100.0%)
Overlong operation (*ScoreT*)	7’187 (12.3%)	9’025 (42.1%)	2’537 (12.7%)
ASA levels 3/4/5	16’777 (28.7%)	9’701 (45.2%)	1’095 (5.5%)
Minimal invasive	24’071 (41.2%)		
Endoscopic		12’261 (57.2%)	
Elective surgery	58’355 (99.8%)	15’532 (72.4%)	9’636 (48.4%)
Year			
2017	21’297 (36.4%)	6’902 (32.2%)	7’106 (35.7%)
2018	20’926 (35.8%)	7’350 (34.3%)	6’528 (32.8%)
2019	16’272 (27.8%)	7’193 (33.5%)	6’283 (31.5%)

IQR Inter Quartile Range; ASA American Society of Anesthesiology

Table S2: Patient characteristics of those included in the analysis compared to those from all surgeries

A. Hip and knee arthroplasty

**Characteristic**<br>N (%) / median [IQR]	**Analysis set**	**All surgeries**
Number of surgeries	20’434	58’495
Number of hospitals	41	127
Surgical site infection	205 (1.0%)	564 (1.0%)
Hospital size (beds)		
<200	15960 (78.1%)	45’475 (77.7%)
200-499	2940 (14.4%)	11’504 (18.9%)
500+	1534 (7.5%)	1’966 (3.4%)
Procedure Type		
Knee	6727 (32.9%)	24’711 (42.2%)
Class		
I (clean)	20434 (100.0%)	58’495 (100.0%)
II (potentially contaminated)	-	-
III (contaminated)	-	-
IV (infected)	-	-
Duration of procedure (mins)	76 [59, 97]	80 [63, 102]
Age (years)	70 [61, 76]	70 [61, 76]
Sex – female	10933 (53.5%)	32’226 (55.1%)
Overlong operation (*ScoreT*)	2096 (10.3%)	7’187 (12.3%)
ASA levels 3/4/5	5957 (29.2%)	16’777 (28.7%)
Minimal invasive	10123 (49.5)	24’071 (41.2%)
Elective surgery	20388 (99.8%)	58’355 (99.8%)
Year		
2017	7212 (35.3%)	21’297 (36.4%)
2018	7288 (35.7%)	20’926 (35.8%)
2019	5934 (29.0%)	16’272 (27.8%)

Pfeiffer Y, *et al. BMJ Open* 2023; 13:e066514. doi: 10.1136/bmjopen-2022-066514

B. Colorectal procedures

**Characteristic**<br>N (%) / median [IQR]	**Analysis set**	**All surgeries**
Number of surgeries	8’321	21’445
Number of hospitals	28	126
Surgical site infection	1’301 (15.6%)	2’973 (13.9%)
Hospital size (beds)		
<200	2’442 (29.3%)	8’622 (40.2%)
200-499	3’115 (37.4%)	7’970 (37.2%)
500+	2’764 (33.2%)	4’853 (22.6%)
Procedure Type		
Rectal	396 (4.8%)	888 (4.1%)
Class		
I (clean)	0 (0.0%)	0 (0.0%)
II (potentially contaminated)	5’641 (67.8%)	14’835 (69.2%)
III (contaminated)	995 (12.0%)	2’548 (11.9%)
IV (infected)	1’685 (20.2%)	4’062 (18.9%)
Duration of procedure (mins)	155 [115, 213]	165 [120, 223]
Age (years)	69 [57, 77]	69 [58, 77]
Sex – female	4’076 (49.0%)	10’849 (50.6%)
Overlong operation (*ScoreT*)	3’131 (37.6%)	9’025 (42.1%)
ASA levels 3/4/5	3’982 (47.9%)	9’701 (45.2%)
Endoscopic	4556 (54.8%)	12’261 (57.2%)
Elective surgery	5831 (70.1%)	15’532 (72.4%)
Year		
2017	2’621 (31.5%)	6’902 (32.2%)
2018	2’799 (33.6%)	7’350 (34.3%)
2019	2’901 (34.9%)	7’193 (33.5%)

Pfeiffer Y, *et al. BMJ Open* 2023; 13:e066514. doi: 10.1136/bmjopen-2022-066514

C. Cesarean sections

**Characteristic**<br>N (%) / median [IQR]	**Analysis set**	**All surgeries**
Number of surgeries	4’346	19’917
Number of hospitals	11	49
Surgical site infection	89 (2.0%)	405 (2.0%)
Hospital size (beds)		
<200	2429 (55.9%)	13’991 (70.2%)
200-499	1128 (26.0%)	5’137 (25.8%)
500+	789 (18.2%)	789 (4.0%)
Class		
I (clean)	0 (0.0%)	0 (0.0%)
II (potentially contaminated)	3313 (76.2%)	14’281 (71.7%)
III (contaminated)	993 (22.8%)	5’235 (26.3%)
IV (infected)	40 (0.9%)	401 (2.0%)
Duration of procedure (mins)	42 [33, 52]	40 [31, 51]
Age (years)	33 [30,36]	33 [30, 37]
Overlong operation (*ScoreT*)	563 (13.0%)	2’537 (12.7%)
ASA levels 3/4/5	244 (5.6%)	1’095 (5.5%)
Elective surgery	2’119 (48.8%)	9’636 (48.4%)
Year		
2017	1276 (29.4%)	7’106 (35.7%)
2018	1’520 (35.0%)	6’528 (32.8%)
2019	1550 (35.7%)	6’283 (31.5%)

Pfeiffer Y, *et al. BMJ Open* 2023; 13:e066514. doi: 10.1136/bmjopen-2022-066514

Patient level analysis

Table S3: Uni- and multivariable estimates from the fitted logistic regression models

A. Hip and knee arthroplasty

**Endpoint:**	Univariable		Multivariable	
**SSI (0/1)**	OR (95% CI)	p-value	OR (95% CI)	p-value
Sex - female	0.8 [0.6, 1.0]	0.05	NS	
Age (in 10 year steps)		NS	-	
ASA – levels 3/4/5	2.2 [1.6, 3.0]	<0.001	2.2 [1.5, 3.0]	<0.001
Overlong operation (“scoreT”)	1.8 [1.1, 3.0]	0.02	1.8 [1.1, 2.9]	0.03
Class		nE	-	
Minimally invasive		NS		
Operation type - Knee (reference Hip)		NS		
Hospital size (number of beds)				
<200	1 (reference)			
200-500	1.5 [0.8, 2.8]	NS		
500+	2.0 [1.0, 4.0]	0.05	NS	
Safety climate (per hospital)		NS	-	
Teamwork (per hospital)		NS	-	
% managerial role (per hospital)		NS	-	
% physician (per hospital)		NS	-	

NS: not significant at the 5% level; nE: not estimated

Pfeiffer Y, *et al. BMJ Open* 2023; 13:e066514. doi: 10.1136/bmjopen-2022-066514

B. Colorectal procedures

**Endpoint:**	Univariable		Multivariable	
**SSI (0/1)**	OR (95% CI)	p-value	OR (95% CI)	p-value
Sex - female	0.8 [0.7, 0.9]	0.005	0.8 [0.7, 1.0]	0.03
Age (in 10 year steps)		NS	-	
ASA – levels 3/4/5	1.7 [1.4, 2.0]	<0.001	1.3 [1.1, 1.6]	0.002
Overlong operation (“scoreT”)	1.5 [1.3, 1.8]	<0.001	1.4 [1.2, 1.7]	<0.001
Class				
II clean-contaminated	1 (reference)			
III contaminated	1.0 [0.8, 1.3]	NS	NS	
IV infected	1.7 [1.4, 2.0]	<0.001	1.3 [1.1, 1.6]	0.01
Endoscopic	0.5 [0.4, 0.6]	<0.001	0.5 [0.4, 0.6]	<0.001
Operation type - rectal	1.3 [1.0, 1.7]	0.07	1.3 [1.0, 1.7]	0.05
Hospital size (number of beds)		NS		
<200				
200-500				
500+				
Safety climate (per hospital)		NS		
Teamwork (per hospital)		NS		
% managerial role (per hospital)	1.0 [1.0, 1.0]	0.1		NS
% physician (per hospital)		NS		

B. Cesarean sections

**Endpoint:**	Univariable		Multivariable	
**SSI (0/1)**	OR (95% CI)	p-value	OR (95% CI)	p-value
Age (in 10 year steps)	0.7 [0.5, 1.0]	0.05		NS
ASA – levels 3/4/5		NS		
Overlong operation ("scoreT")		NS		
Class				
II clean-contaminated	1 (reference)		1 (reference)	
III contaminated	NS		NS	
IV infected	4.4 [2.3, 8.6]	0.002	3.5 [1.7, 7.2]	0.02
Hospital size (number of beds)				
<200	1 (reference)		1 (reference)	
200-500	NS		NS	
500+	2.7 [1.9, 3.9]	<0.001	2.8 [2.0, 4.0]	0.001
Safety climate (per hospital)		NS		
Teamwork (per hospital)		NS		
% managerial role (per hospital)		NS		
% physician (per hospital)	1.0 [1.0, 1.0]	0.02		NS

Pfeiffer Y, *et al. BMJ Open* 2023; 13:e066514. doi: 10.1136/bmjopen-2022-066514

Table M1 Descriptive statistics of complete cases and following multiple imputation for knee and hip surgeries

	**Hip and knee**		
median [IQR]	Complete cases	Multiply imputed (K=20)	p-value
Safety climate (%)	45.5 [12.5, 83.3]	50 [16, 87]	0.09
% leaders	20% [0, 50]	25% [6, 55]	<0.001
% physician	30% [0, 100]	44% [0, 100]	<0.001
Teamwork (%)	50% [14, 89]	55 [10, 92]	0.19
% leaders	20% [0, 50]	25% [6, 55%]	<0.001
% physician	33% [0, 100]	44% [0, 100]	<0.001

Table M2: Comparison of estimates from the fitted weighted linear model for knee and hip surgeries

	Hip and knee<br>slope estimate (95% CI), p-value	
Endpoint	Complete cases	Multiply imputed (K=20)
% safety climate regressed on infection rate1	-100 (-215, 15), p=0.10	-182 (-296, -68), p=0.003
% Teamwork regressed on infection rate1	-59 (-186, 67.7), p=0.37	-98 (-218, 22), p=0.11

[1]square root of the infection rate

Pfeiffer Y, *et al. BMJ Open* 2023; 13:e066514. doi: 10.1136/bmjopen-2022-066514
