# Supplementary material for: Are cross-sectional safety climate survey results in operating room staff associated with the surgical site infection rates in Swiss hospitals?
Source: BMJ Open. 2023 Apr 19;13(4):e066514. doi: 10.1136/bmjopen-2022-066514 (PMC10124250; doi:10.1136/bmjopen-2022-066514)
Supplement: Supplementary data [file bmjopen-2022-066514supp002.pdf]

APPENDIX

	Recoded
**SCS Safety Climate Scale (SCS)**	
(1) The culture of this clinical area makes it easy to learn from the mistakes of others.	
(2) Medical errors** are handled appropriately in this clinical area. ** Medical error is defined as any mistake in the delivery of care, by any healthcare professional, regardless of outcome.	
(3) The senior leaders in my hospital listen to me and care about my concerns.	
(4) The physician and nurse leaders in my area listen to me and care about my concerns.	
(5) I am encouraged by my colleagues to report any safety concerns I may have.	
(6) I know the proper channels to direct questions regarding patient safety.	
(7) Leadership is driving us to be a safety- centered institution.	
(8) I receive appropriate feedback about my performance.	
(9) I would feel safe being treated here as a patient.	
(10) I am satisfied with the availability of clinical leadership (please respond to all three):	
a) Physician;	
b) Nursing;	
c) Pharmacy	
(11) Briefing personnel before the start of a shift (i.e., to plan for possible contingencies) is an important part of patient safety.	
(12) Briefings are common here.	
(13) This institution is doing more for patient safety now than it did one year ago.	
(14) I believe that most adverse events occur as a result of multiple system failures and are not attributable to one individual's actions.	
(15) The personnel in this clinical area take responsibility for patient safety.	
(16r) Management/leadership does not knowingly compromise safety concerns for productivity.	recoded
(17r) Personnel frequently disregard rules or guidelines that are established for this clinical area.	recoded
(18) Patient safety is constantly reinforced as the priority in this clinical area.	
(19r) In this clinical area, it is difficult to discuss errors.	recoded
(20) My suggestions about safety would be acted upon if I expressed them to management.	
**TWC Teamwork Climate Scale (TWC)**	
(1) Nurse input is well received in this clinical area.	
(2) In this clinical area, it is difficult to speak up if I perceive a problem with patient care.	recoded
(3) Disagreements in this clinical area are resolved appropriately (i.e., not who is right, but what is best for the patient).	
(4) I have the support I need from other personnel to care for patients.	
(5) It is easy for personnel here to ask questions when there is something that they do not understand.	
(6) The physicians and nurses here work together as a well-coordinated team.	
