# Supplementary material for: Are cross-sectional safety climate survey results in operating room staff associated with the surgical site infection rates in Swiss hospitals?
Source: BMJ Open. 2023 Apr 19;13(4):e066514. doi: 10.1136/bmjopen-2022-066514 (PMC10124250; doi:10.1136/bmjopen-2022-066514)
Supplement: Author's manuscript [file bmjopen-2022-066514.draft_revisions.pdf]

BMJ Open is committed to open peer review. As part of this commitment we make the peer review history of every article we publish publicly available.

When an article is published we post the peer reviewers' comments and the authors' responses online. We also post the versions of the paper that were used during peer review. These are the versions that the peer review comments apply to.

The versions of the paper that follow are the versions that were submitted during the peer review process. They are not the versions of record or the final published versions. They should not be cited or distributed as the published version of this manuscript.

BMJ Open is an open access journal and the full, final, typeset and author-corrected version of record of the manuscript is available on our site with no access controls, subscription charges or pay-per-view fees (http://bmjopen.bmj.com).

If you have any questions on BMJ Open's open peer review process please email info.bmjopen@bmj.com

BMJ Open

Are surgical site infection rates associated with safety climate as assessed in a survey of operation room staff?

Journal:	*BMJ Open*
Manuscript ID	bmjopen-2022-066514
Article Type:	Original research
Date Submitted by the Author:	31-Jul-2022
Complete List of Authors:	Pfeiffer, Yvonne; Stiftung fur Patientensicherheit<br>Atkinson, Andrew; Inselspital University Hospital Bern, Department of Infectious Diseases<br>Maag, Judith; Inselspital University Hospital Bern, Department of Infectious Diseases; Swissnoso, National Center for Infection Control, Bern, Switzerland<br>Lane, Michael; Parkland Health and Hospital System<br>Schwappach, David; Universität Bern, Institute of Social and Preventive Medicine<br>Marschall, Jonas; Inselspital Universitatsspital Bern, Department of Infectious Diseases; Washington University School of Medicine in Saint Louis, Division of Infectious Diseases
Keywords:	EPIDEMIOLOGY, Health & safety < HEALTH SERVICES ADMINISTRATION & MANAGEMENT, Risk management < HEALTH SERVICES ADMINISTRATION & MANAGEMENT, Infection control < INFECTIOUS DISEASES, PUBLIC HEALTH, Adult surgery < SURGERY

SCHOLARONE™
Manuscripts

I, the Submitting Author has the right to grant and does grant on behalf of all authors of the Work (as defined in the below author licence), an exclusive licence and/or a non-exclusive licence for contributions from authors who are: i) UK Crown employees; ii) where BMJ has agreed a CC-BY licence shall apply, and/or iii) in accordance with the terms applicable for US Federal Government officers or employees acting as part of their official duties; on a worldwide, perpetual, irrevocable, royalty-free basis to BMJ Publishing Group Ltd ("BMJ") its licensees and where the relevant Journal is co-owned by BMJ to the co-owners of the Journal, to publish the Work in this journal and any other BMJ products and to exploit all rights, as set out in our licence.

The Submitting Author accepts and understands that any supply made under these terms is made by BMJ to the Submitting Author unless you are acting as an employee on behalf of your employer or a postgraduate student of an affiliated institution which is paying any applicable article publishing charge ("APC") for Open Access articles. Where the Submitting Author wishes to make the Work available on an Open Access basis (and intends to pay the relevant APC), the terms of reuse of such Open Access shall be governed by a Creative Commons licence – details of these licences and which Creative Commons licence will apply to this Work are set out in our licence referred to above.

Other than as permitted in any relevant BMJ Author's Self Archiving Policies, I confirm this Work has not been accepted for publication elsewhere, is not being considered for publication elsewhere and does not duplicate material already published. I confirm all authors consent to publication of this Work and authorise the granting of this licence.

For peer review only

Title

Are surgical site infection rates associated with safety climate as assessed in a survey of operation room staff?

Brief Title

SSI rate association with safety climate

Authors

Yvonne Pfeiffer*[1], Dr. sc.; Andrew Atkinson[2], PhD; Judith Maag[2,3] MSc., M.A., MPH; Michael A Lane, MD, MPHS[5]; David L. B. Schwappach[4 +] PhD, MPH.; Jonas Marschall[2,3,6 +], MD.

[1]Swiss Patient Safety Foundation, Zurich, Switzerland; Asylstr. 77, 8032 Zurich, Switzerland, Tel. 0041 43 2441480, Fax 0041 43 2441481, E-mail: ypfeifferdrsc@gmail.com

[2]Department of Infectious Diseases, Bern University Hospital, Inselspital, University of Bern, Bern, Switzerland.

[3]Swissnoso, National Center for Infection Control, Bern, Switzerland

[4]Institute of Social and Preventive Medicine (ISPM), University of Bern, Switzerland

[5]Parkland Health, Dallas, Texas, United States

[6] Division of Infectious Diseases, Washington University School of Medicine, Saint Louis, MO, United States

[+]These authors contributed equally

Corresponding author: Yvonne Pfeiffer, Swiss Patient Safety Foundation, Zurich, Switzerland; Asylstr. 77, 8032 Zurich, Switzerland, Tel. 0041 43 2441480, Fax 0041 43 2441481, E-mail: ypfeifferdrsc@gmail.com

WORD COUNT: 3588

For peer review only

STRUCTURED ABSTRACT

Objectives. The aim of this study was to investigate the association between surgical site infections (SSI), a major source of patient harm, and safety and teamwork climate. Prior research has been unclear regarding this relationship.

Design. Based on the swiss national SSI surveillance and a survey study assessing a) safety climate and b) teamwork climate, associations were analyzed for three kinds of surgical procedures.

Setting and participants. SSI surveillance data from 20'434 surgeries for hip and knee arthroplasty from 41 hospitals, 8'321 for colorectal procedures from 28 hospitals and 4'346 cesarean sections from 11 hospitals and survey responses from Swiss operating room personnel (N=2769) in 54 acute care hospitals.

Primary and secondary outcomes. The primary endpoint of the study was the 30 day (all types) or 1 year (knee/hip with implants) NHSN-adjusted SSI rate. Its association with climate level and strength was investigated in regression analyses, accounting for respondents' professional background, managerial role, and hospital size as confounding factors.

Results. Plotting climate *levels* against infection rates revealed a general trend with SSI rate decreasing as the safety climate increased, but none of the associations were significant (5% level). Linear models for hip and knee arthroplasties showed a negative association between SSI rate and climate perception (p=0.02). For climate *strength*, there were no consistent patterns, indicating that alignment of perceptions was not associated with lower infection rates. Being in a managerial role and being a physician (vs. a nurse) had a positive effect on climate levels regarding SSI in hip and knee arthroplasties, whereas larger hospital size had a negative effect.

Conclusions. This study suggests a possible negative correlation between climate level and SSI rate, while for climate strength no associations were found. Future research should study safety climate more specifically related to infection prevention measures to establish clearer links.

For peer review only

STRENGTHS AND LIMITATIONS OF THIS STUDY

- This study used national surgical site infections surveillance data of high quality, also including infection events after discharge from the hospital.
- For modelling purposes, safety climate and teamwork climate outcomes, as well as level and strength of climate perceptions, were differentiated.
- Including three different types of surgical procedures allowed for controlling for influences stemming from inherent variations in risk profiles depending on the kind of surgery.
- Potential underreporting of surgical site infections may have had a biasing effect on the analysis.
- Climate could only be aggregated to hospital level and not team or unit level, due to the ad hoc formation of surgery teams. Aggregation on the hospital level (and weighting) could conceal certain trends.

KEYWORDS

Surgical Site Infection; Surgical Site Infection Rate; Safety Climate; Teamwork Climate; Safety Attitude Questionnaire; Operating Room; Operating Room Personnel; Infection Prevention and Control.

For peer review only

Introduction

Healthcare-associated infections are a major concern for patient safety. Specifically, surgical site infections (SSI) are considered the most common healthcare-associated infection, occurring in 2-5% of inpatient surgeries and represent an economic burden to the healthcare system [1]. SSI rates may be reduced effectively by performing adequate infection prevention measures, such as the use of antibiotic prophylaxis or skin disinfection prior to incision. For example, the adherence to a standardized bundle of prevention measures reduced SSI after colon surgery in a retrospective cohort study, thus improving patient safety [2]. In order for healthcare workers to perform safety-relevant behaviors, safety climate is thought to play an important role. Specifically, compliance with infection prevention measures is thought to be influenced by the prevailing safety climate [3,4], however, only with mixed supporting evidence so far [5]. Similarly, safety culture and climate have been used as an explanatory factor for failed implementation of preventive interventions that were successful in the original studies [6].

"Safety climate" encompasses shared perceptions related to safety policies, procedures, and practices [7] and is expected to guide the safety behavior of workers. There is limited evidence from prior research indicating that safety climate may be linked to healthcare-associated infections, and SSI in particular: Fan et al. [8] found that nine of twelve safety climate dimensions were associated with SSI rates after colon surgery. To evaluate a program reducing SSIs after colorectal surgery in Hawaiian hospitals, Lin et al. [9] assessed safety climate at baseline and after implementation of the program; however, no consistent patterns of association of (change in) SSI rate with (change in) safety climate dimensions could be identified.

Based on the broad coverage and longitudinal assessment of SSI captured by the Swiss surveillance system, this study relates SSI rates to safety climate scores. The study aims were to investigate a) whether better safety climate *level* and higher safety climate *strengths* are associated with lower SSI rates, and b) whether these relationships are also true for teamwork climate scores.

For peer review only - http://bmjopen.bmj.com/site/about/guidelines.xhtml

A prior study of our group showed that infection rates may vary with hospital size, meaning that larger hospitals have higher rates, confounded by their case mix and type of surgeries investigated [10]. Therefore, hospital size was considered in our analyses. That study also investigated whether quality of surveillance, assessed by external audits, has an influence on the measured SSI rate, and we considered "quality of surveillance" as assessed with the audit score here, too: hospitals conducting a thorough surveillance demonstrate their ability to implement and follow relevant procedures, which we hypothesized may also be related to safety climate. Three types of procedures were included in the study: colorectal surgery, cesarean section, and knee/hip arthroplasties. They were chosen for being the procedures for which the largest number of institutions report SSI rates in Switzerland.

In order to carefully evaluate safety climate as a characteristic of a unit or organization [11], we assessed climate *strength* in addition to the safety climate *level* indicating positive or negative climate evaluations: Climate strength indicates how aligned the individual climate perceptions are with each other in a given unit, and is considered a predictor for safety outcomes [12,13] that can provide valuable insights. While the level of safety climate scores may not change or differ, their strength, indicating consensus or divergence of perceptions regarding safety climate, may do so [14,15].

As prior research exploring the relationship between safety climate and SSI rates has advocated [9], we used one composite safety climate scale. We added the scale 'teamwork climate' because teamwork is important for safe outcomes in surgeries, and it has been linked to healthcare-associated infections in the NICU setting before [16].

Prior research has also shown that those in managerial positions tend to rate safety climate higher than frontline healthcare workers [17–19], and also that the professional background (here "nurse" or "physician") has an influence on safety climate perceptions [17,18]. This has been traced back to various explanations, such as the existence of different management structures, subcultures, or work demands in these two groups. Therefore, in this study, the managerial role and professional background were taken into account when analyzing the relationship with infection rates.

For peer review only - http://bmjopen.bmj.com/site/about/guidelines.xhtml

Methods

Sample and missing data

All hospitals and hospital networks providing acute care and participating in the SSI surveillance module of Swissnoso were invited (N= 143) to participate in the study, of which N=54 institutions agreed. Among these, all operation room (OR) personnel received the survey. An exact response rate cannot be estimated, as total numbers of healthcare workers in operating rooms were not retrievable. However, we know that 38% of all paper surveys sent out were completed. Survey participation was considered informed consent.

After exclusion of participants with demographic answers that were inconsistent, e.g., indicating that they worked as physician and as nurse at the same time (7 participants), and those with less than 32 (from 52) responses (36 participants), we had a sample of N = 2769 (see figure S1).

For SSI rates, we selected adult (≥16 years) patients with complete follow-up information in 2017-19 (3 years) for elective hip and knee arthroplasties (58'495 procedures, 127 hospitals), colorectal surgeries (21'445 procedures, 126 hospitals), and cesarean sections (19'917 procedures, 49 hospitals) from the Swissnoso database (extraction performed on 30.9.2020). We assumed this would provide an adequate summary of the infection rates at the time of the survey in 2019. Combining survey data with infection rates meant limiting the sample size, as not all surgery types are reported by every hospital. Equally, for us to match survey responses with persons that work in the surveilled surgery types, each survey respondent had to list the type of surgeries they participate in. Data was collected on the patient and participant level, and then aggregated on hospital level. The resulting analysis set consisted of 20'434 surgeries for hip and knee arthroplasty from 41 hospitals, 8'321 for colorectal procedures from 28 hospitals and 4'346 cesarean sections from 11 hospitals (see figure S1 and table S1).

In addition to the complete case analysis, we multiply imputed missing responses from individual survey participants using predictive mean matching with the 10 nearest neighbors as donor pool in the algorithm [20].

For peer review only - http://bmjopen.bmj.com/site/about/guidelines.xhtml

Safety Climate and Teamwork Climate Measures

In order to assess climate, the safety climate (*SCS*, 22 items) and teamwork climate (*TWC*, 6 items) scales of the Safety Attitudes Questionnaire (SAQ)[21] were used (see appendix). The internal consistency of the *SCS* was high: Cronbach Alpha = .89; for *TWC* it was slightly lower with Cronbach Alpha = .78, but still acceptably high.

The survey was sent out in the three national languages: A prior study had developed Swiss versions in German and French [22], and the Italian version was developed by applying a translation and back-translation process using different translators, blinded to the original items. Any differences were resolved, and the items tested in two sites by OR personnel for understandability and correctness. Two different measures were used in the analyses: safety and teamwork climate *level* and safety and teamwork climate *strength*. Climate level was measured as percent positive responses (PPR) per hospital. Percentage of positive respondents was calculated counting each respondent's score as positive for their unit if their mean score on the scale was 4.0 or greater (from a possible score of 1-5), in line with Tawfik et al's approach. [15]. Intra-hospital correlation of the climate level scores (*rwg*, a measure of within-group agreement) was checked in order to justify aggregation on the hospital level: all *rwgs* were above .66, with their median being .78 (*SCS*) and .81 (*TWC*), respectively, indicating moderate to strong agreement [23]. In line with prior research, climate strength was evaluated using the standard deviation of mean climate level scores per hospital [15,24,25].

Assessment and adjustment of SSI rates and incorporation of audit scores

Analyses were conducted separately for the surgery types, because SSI rates are not comparable between specialties since certain procedures are more prone to SSI than others. The primary endpoint of the study was the 30 day (all types) or 1 year (hip/knee with implants) NHSN (National Healthcare Safety Network)-adjusted rate of infection [26], and this was assessed in all patients age 16 years and older for the respective hospitals in the 3-year study period. For hip/knee surgeries, only class I

For peer review only - http://bmjopen.bmj.com/site/about/guidelines.xhtml

("clean") surgeries were included, but all classes were included for colorectal surgeries and cesarean sections.

Despite the sophisticated surveillance system employed in Switzerland, the accuracy of the SSI rates may be affected by underreporting. Therefore, audit scores from on-site quality audits were also taken into account. These hospital audit scores indicate how thoroughly the SSI surveillance is conducted in a given hospital, with higher audit scores were linked to higher rates of reported SSI for certain surgeries [10]. Audit scores range from 0 to 50, and cover aspects such as how structured the data assessment is, how well trained the persons conducting it are, among others [27].

Data Analyses

The primary analysis was based on complete cases only and considered the correlation between the SSI rate, aggregated on the hospital level, and the safety climate and teamwork climate.

Categorical variables are shown with the number per category (N) along with the appropriate percentage of the total, with continuous variables summarized as median and inter-quartile range. Group differences were investigated using the Kruskal-Wallis non-parametric test.

We began by visually checking for associations by plotting safety and teamwork climate scores (level and strength) against the standardized infection rates for each procedure type, stratified by hospital size (<200, 200-499, and 500+ beds).

Weighted linear models were fitted with the respective safety climate endpoint as dependent variable, and as independent variables, the SSI rate, percentage of managerial (vs. non-managerial) roles and percentage of physicians (vs. nurses) having responded to the survey in a hospital for the specific surgery type, the hospital size and hospital type (university, regional, or "other"). The number of surgeries in a given hospital was used for the weighting in the models.

Those variables statistically significant at the 10% level in univariable analyses were included in the multivariable analysis with forwards selection and backwards deletion using the AIC (Akaike information criterion) to determine the most parsimonious model.

For peer review only - http://bmjopen.bmj.com/site/about/guidelines.xhtml

Patient and public involvement

To stay safe from SSI is important for patients undergoing surgery, and to study the role of safety climate thus can offer important avenues for improvement. From the time when the project was funded, the public was informed on the website of Swissnoso (National Center for Infection Control, Bern, Switzerland) and on the website and in the newsletter of the patient safety foundation. The patients were not involved in the design and conduct of the study.

Results

Mean safety climate level scores were very similar between the healthcare workers involved in the three procedure types (c-section 43.3, inter-quartile range [0.0, 66.8], hip/knee median PPR 45.5 [12.5, 83.3], colorectal 48.1 [4.0, 100.0]), but as the ranges show, with large variability between hospitals. As expected, standardized infection rates mirrored the underlying population and surgery type differences between the surgery types (hip/knee median 1.0% [0.0, 3.0], colorectal 16.0% [7.0, 33.0], c-section 2.0% [1.0, 4.0], top part of Table 1, supplementary material figure S2). The median number of survey respondents per hospital was deemed adequate, although there were some imbalances due to low numbers in some hospitals. Approximately 20% of responders had a managerial role and approximately 30% were physicians (as opposed to nurses), although the percentage of physicians was somewhat lower for cesarean sections. Interestingly, despite the comparable safety climate level scores, teamwork climate level scores indicated a slightly lower median value for hip/knee compared to the other two procedure types (48.1 compared to 55.6 and 54.3 respectively, bottom part of Table 1).

For peer review only

For peer review only - http://bmjopen.bmj.com/site/about/guidelines.xhtml

Table 1: NNIS adjusted SSI rate compared to safety climate and teamwork scores, stratified by surgery type (NNIS: National Nosocomial Infections Surveillance)

N (%) / median [IQR]	**Hip and knee arthroplasty**	**Colorectal**	**Cesarean section**
Safety climate (%)	45.5 [12.5, 83.3]	48.1 [4.0, 100.0]	43.3 [0.0, 66.7]
Number of hospitals	41	28	11
Number of surgeries	20'434	8'321	4'346
NNIS adjusted infection rate	1.0% [0.0, 3.0]	16.0% [7.0, 33.0]	2.0% [1.0, 4.0]
Hospital size (beds)			
<200	27 (65.9%)	13 (46.4%)	8 (72.7%)
200-499	9 (22.0%)	9 (32.1%)	2 (18.2%)
>500	5 (12.2%)	6 (21.4%)	1 (9.1%)
Number of participants	754	493	210
participants per hospital	17 [5, 53]	12 [5, 71]	12 [6, 40]
Percentage leaders	20.0% [0.0, 50.0]	21.4 [0.0, 80.0]	18.2% [5.9%, 50.0%)
Percentage physicians (vs. nurses)	30.0% [0.0, 100.0]	34.5 [0.0, 100.0]	20.0% [0.0, 57.5]
Teamwork (%)	48.1 [4.0, 100.0]	55.6 [7.4, 88.9]	54.3 [0.0, 100.0]
Number of hospitals	44	28	12
Number of surgeries	21'247	8'321	4'824
NNIS adjusted infection rate	1.0% [0.0, 3.0]	16.0% [7.0, 31.0]	2.0% [1.0, 4.0]
Hospital size (beds)			
<200	30 (68.2%)	13 (46.4%)	9 (75.0%)
200-499	9 (20.5%)	9 (32.1%)	2 (16.7%)
>500	5 (11.4%)	6 (21.4%)	1 (8.3%)
Number of participants	861	549	239
Participants per hospital	18 [5, 56]	14 [5, 72]	14 [5, 42]
Percentage leaders	20.0% [0.0, 50.0]	21.4% [0.0, 80.0]	19.4% [5.6, 44.4]
Percentage physicians	33.3% [0.0, 100.0]	34.4% [0.0, 100.0]	22.0% [0.0, 57.4]

For peer review only

Otherwise, the characteristics were very similar to those for the safety climate endpoint. The stratification by hospital size did not reveal any major differences, although these comparisons certainly lacked power in some of the subcategories (figure S3). The SSI data used for the analysis was broadly comparable with that from the underlying Swissnoso population, with little discernible selection bias (table S2).

Plots of the safety endpoints against the standardized SSI rate for each of the surgery types showed generally decreasing trends, in line with our hypothesis that as safety climate and teamwork increase, the SSI rate drops. However, with the exception of the safety climate endpoint for hip and knee

For peer review only - http://bmjopen.bmj.com/site/about/guidelines.xhtml

surgeries (p=0.02 for slope in adjusted linear models, see table 2), these findings were not supported by formal statistical tests (figure 1). Similarly, *strength* of safety climate and teamwork climate, as measured by the standard deviation of the respective metric, was also inconclusive, although once again safety climate showed a promising trend (figure 2).

Table 2: Estimates following fitting of unadjusted and adjusted weighted linear models

A. Safety Climate

Endpoint:<br>% safety climate	**Hip and knee**<br>est., p-value		**Colorectal**<br>est., p-value		**Cesarean section**<br>est., p-value	
	univariable	multivariabl<br>e	univariable	multivariabl<br>e	univariable	multivariabl<br>e
**SSI rate**	- , p=0.05	-, p=0.02	NS	-	NS	-
% managerial role	+, p=0.08	nE	+, p=0.01	-	+, p=0.1	-
% physician	+, p=0.08	+, p=0.03	NS	-	NS	-
Hospital size		NS		-		-
<200	reference		reference		reference	
200-499	NS		NS		NS	
500+	-, p=0.09		NS		NS	
Hospital type		-		-		-
Cantonal hospital	reference		reference		reference	
Others	NS		NS		NS	
University	NS		NS		NS	

For peer review only

For peer review only - http://bmjopen.bmj.com/site/about/guidelines.xhtml

B. Teamwork

Endpoint: % safety climate	**Hip and knee** est., p-value		**Colorectal** est., p-value		**Cesarean section** est., p-value	
	univariable	multivariable	univariable	multivariable	univariable	multivariable
SSI rate	NS	-	NS	-	NS	-
% managerial role	+, p=0.1	nE	+, p=0.005	-	+, p=0.1	-
% physician	+, p=0.07	+, p=0.04	+, p=0.03	-	NS	-
Hospital size				-		-
<200	reference	reference	reference		reference	
200-499	NSNS	NS	NS		NS	
500+		NS	NS		NS	
Hospital type				-		-
Kantonspital	reference	reference	reference		reference	
Others	NS	NS	NS		NS	
University	-, p=0.08	-, p=0.04	NS		NS	

est. estimate; CI confidence interval; **+** increasing trend (ie positively correlated), **-** decreasing trend (i.e., negative correlated); NS not significant at the 5% level; nE not estimated since managerial role and physician are collinear – we include "% managerial role"; hospital size and hospital type are collinear, we include hospital size

Furthermore, univariable linear models indicated that higher percentages of both managerial roles and physicians were associated with higher teamwork scores for hip/knee and colorectal surgeries. For patient-level analyses, please see table S3.

Subgroup analysis

In a subgroup analysis of hip/knee surgeries, in which a marginal association with SSI rate was apparent, we investigated whether larger hospitals are, in some sense, more critical about their own work processes, and hence have lower safety climate level scores. The right hand side of figure 3 confirms there is no difference in safety climate level scores based on hospital size (<200 beds, 200+ beds), despite a significantly higher SSI rate in larger hospitals (p=0.02). Factoring in the audit score, a measure of the SSI surveillance thoroughness but here used as a proxy for "adherence to safety-relevant protocols and standards", revealed a slightly lower median safety climate score in those with higher audit scores (i.e. more thorough adherence) for larger (200+ beds) compared to smaller hospitals (figure 4, far right set of box plots).

For peer review only

For peer review only - http://bmjopen.bmj.com/site/about/guidelines.xhtml

Missing data analysis

We multiply imputed 20 complete data sets (N=2'703 survey participants, compared to N=754 in the complete case analysis) making the missing at random assumption, including responses from all questions (safety climate and teamwork climate), along with the relevant covariates from table 1 in the imputation model, and then repeated the primary analysis for knee and hip arthroplasties. We compared descriptive statistics and the linear model estimates with those from the complete case analysis. Following multiple imputation (MI) there was an increase in physician (and "persons with managerial role") participation, indicating that physicians were less likely to complete the questionnaire (supplementary material table M1, e.g., 30% to 44% for physicians for the safety climate endpoint, $p<0.001$). Otherwise, the results were similar with slight, but not statistically significant, increases in safety climate and teamwork level score for the multiply imputed data (table M2).

Discussion

The idea that a good safety climate in an institution is associated with good safety outcomes for its patients is prominent in health services research. By their incidence and susceptibility to prevention measures, SSI rates are a suitable safety outcome to be linked to measurements of safety climate. As the Swiss assessment of SSI is of high quality (including infections becoming obvious after discharge from the hospital and the surveillance quality being audited on a biannual basis), this study represented a good opportunity to better understand the relationship between safety climate and safety outcomes. Indeed, this study revealed some associations between climate level and SSI rate in the expected direction, that is, the higher the safety climate, the lower the SSI rate. However, only for hip and knee surgeries was a formal statistical association observed. As implant surgery is "cleaner" than the other surgery types, it is probable that the diligence of adhering to preventive measures is better reflected in the respective infection outcomes; this may explain why the association was most salient for these surgeries. Overall, however, this study did not empirically support the expected associations between safety and teamwork climate among Swiss OR personnel and SSI rates in a strong way.

For peer review only - http://bmjopen.bmj.com/site/about/guidelines.xhtml

To investigate whether larger organizations are more safety-conscious and therefore have a more critical view of their own environment, we compared SSI rates and safety climate levels between larger and smaller hospitals for the arthroplastic surgery sample, but there was no difference in climate that could have corroborated this idea. In a further step, we explored whether the thoroughness of the SSI surveillance process was associated with safety climate for hip/knee surgeries (in which a marginal association with SSI rate was encountered): Larger hospitals in fact tended to have lower safety climate scores, the higher their surveillance audit scores were. Here, we are interpreting a high audit score as a marker for the ability to comply with external regulations and to follow through in implementing procedures. A hospital's workforce that is capable of maintaining a high-quality surveillance and reporting process according to national requirements may therefore be less likely to report overly optimistic safety climate scores. Such an organization obviously does much to adhere to standards and protocols, and staff may be enabled to provide a more realistic, or even critical, evaluation of teamwork and safety climate. The combination of low safety climate scores with high audit scores could therefore point to larger hospitals having a higher 'sensitivity to their operations', which is an important feature of high-reliability organizations [28].

Investigating the role of climate *strength* did not yield coherent and easily interpretable results, neither for safety climate nor for teamwork climate. We expected higher strength (i.e., higher agreement of climate perceptions) to be reflected in lower SSI rates. When plotting the strength scores against the infection rates, this assumption was confirmed for knee and hip surgeries as well as for cesarean sections. However, for colorectal surgeries and for the relationship between teamwork climate and cesarean section SSI rates, the direction of the association was contrary to our expectation. As we evaluated climate strengths per hospital, these mixed findings might be traced back to the frequent ad hoc formation of surgical teams which makes it hard to define team boundaries and to develop aligned perceptions of climate.

Having a managerial role or being a physician had a positive influence on climate levels for two surgery types. This result is in line with earlier research indicating that persons with managing roles have a more positive view on safety climate [19,29,30], and with reports of physicians evaluating safety climate more optimistically than nurses [31], although that difference is not as clear as for managerial

For peer review only

roles [32]. Future studies will need to shed light on the origins of differences in safety climate perceptions and their potential effects on safety outcomes.

We decided to use the safety climate scale *and* the teamwork climate scale of the SAQ [21,22], as the latter specifically covers nurse-physician collaboration in two items. Differentiating the composite safety climate dimension from the specific teamwork climate dimension revealed some nuances in the results, but overall, it did not provide particularly new insights. While Fan et al. [33] in their prior study in colon surgery investigated different climate dimensions, Lin et al. [9] argued for using composite scores, a suggestion that is supported by our results.

Limitations

Climate could only be aggregated to hospital level, due to the usual ad hoc formation of surgery teams. Respondents' identification with a different group than with their hospital was not assessed and therefore some of the real-world complexity of climate perceptions may have been lost.

Both the number of patients and hospitals included in the study provide sufficient statistical power for investigating the trends in question. However, aggregation on the hospital level (and weighting) could conceal certain trends. However, a post-hoc patient level analysis without hospital level aggregation confirmed the results from the main analysis.

For the analyses following multiple imputation of missing data, the slope estimate when regressing safety climate on the SSI rate for hip/knee arthroplasties turned out to be statistically significant ($p=0.003$), which was not the case for the analysis including only the complete cases. We chose to include the more conservative estimates from the complete case analysis in the results. Therefore, we take the opportunity to highlight this potential bias, and the considerable effect of including more data from physicians in the analysis.

In adding to the inconsistent and in many cases non-significant [34,35] findings of the relationship of climate level and strength with various safety outcomes, such as medication errors [36], patient evaluations [35], length of stay [15], and healthcare-associated infections [36], this study raises two areas for future research: First, surveys may not fully assess the perceptions that are actually relevant for performing specific safety behaviors such as adhering to SSI prevention measures. As Meeks et al. [5]

For peer review only - http://bmjopen.bmj.com/site/about/guidelines.xhtml

reported, for achieving compliance with SSI prevention measures, specific interventions were needed and having a good safety climate was "simply not enough". Groves [37] suspected that safety climate surveys only assess the declared values in an organization, which is not necessarily what guides an individual's behaviors. For SSI rates, this would mean integrating perceptions around performing the relevant SSI prevention measures such as administering the correct antibiotic prophylaxis in a timely fashion, for example. Applying additional qualitative approaches such as observations and interviews to learn more about the attitudes and perceptions around infection prevention measures could potentially be a fruitful avenue [38]. Secondly, to study the influence of safety climate on outcomes, it is necessary to make sure that the outcome is malleable to a large degree by safety-conscious behavior. The fact that our results are clearer for arthroplastic surgery underscores this link. Thus, outcomes should be chosen that are highly sensitive to correct infection prevention behaviors and have as few confounding influences as possible.

Conclusion

There is a growing body of research developed under the assumption that safety culture needs to be improved before or concurrently with the introduction of infection prevention measures or when a specific infection prevention measure is not showing success. However, this study adds to the inconsistent results around associations between safety climate and healthcare-associated infection rates. Future studies will need to apply other safety climate assessment methods, which should be tied more closely to the actual behavior regarding infection prevention measures. Thus, using an outcome measure indicating whether prevention measures have been adhered to will be an important way to learn more about the link between safety climate and safety outcomes.

For peer review only - http://bmjopen.bmj.com/site/about/guidelines.xhtml

CONTRIBUTORSHIP STATEMENT

Yvonne Pfeiffer: Acquisition of data; analysis and interpretation of data; drafting of manuscript; critical revision of manuscript

Andrew Atkinson: Analysis and interpretation of data; drafting of manuscript; critical revision of manuscript

Judith Maag: Acquisition of data; analysis and interpretation of data; critical revision of manuscript

Michael A Lane: Study conception and design; critical revision of manuscript

David L. B. Schwappach: Study conception and design; analysis and interpretation of data; drafting of manuscript; critical revision of manuscript

Jonas Marschall: Study conception and design; analysis and interpretation of data; critical revision of manuscript.

COMPETING INTERESTS STATEMENT

The authors declare no competing interests.

FUNDING

This work was supported by a research grant from the Swiss National Science Foundation, Nr.: 179500 (Project "Understanding the drivers of surgical site infection: Investigating and modelling the Swissnoso surveillance data (*Watussi*)"). The funding source was not involved in study design, collection, analysis, and interpretation of data gathered nor in the writing of or decision to submit the manuscript.

For peer review only - http://bmjopen.bmj.com/site/about/guidelines.xhtml

DATA SHARING STATEMENT

Data supporting this study will be available in anonymized form for additional research under the specifications set forth by Swissnoso and the ANQ (Swiss National Association for Quality Development in Hospitals and Clinics). In principle, any researcher can submit a proposal to use Swissnoso data (including complementary data used in this study), which will be reviewed by the Swissnoso board.

ETHICS APPROVAL

The Cantonal Ethics Committee (Bern, Project ID 2019-00294) approved the study.

For peer review only

For peer review only - http://bmjopen.bmj.com/site/about/guidelines.xhtml

References

1. Ban KA, Minei JP, Laronga C, et al. American College of Surgeons and Surgical Infection Society: Surgical Site Infection Guidelines, 2016 Update. *J Am Coll Surg*. 2017;224:59–74.

2. Guerrero MA, Anderson B, Carr G, et al. Adherence to a standardized infection n reduction bundle decreases surgical site infections after colon surgery: a retrospective cohort study on 526 patients. *Patient Saf Surg*. 2021;15:15.

3. De Bono S, Heling G, Borg MA. Organizational culture and its implications for infection prevention and control in healthcare institutions. *J Hosp Infect*. 2014;86:1–6.

4. Sakamoto F, Tomoko S, Saint S, et al. Health care-associated infection prevention in Japan: The role of safety culture. *Am J Infect Control*. 2014;42:888–893.

5. Meeks DW, Lally KP, Carrick MM, et al. Compliance with guidelines to prevent surgical site infections: As simple as 1-2-3? *Am J Surg*. 2011;201:76–83.

6. Dixon-Woods M, Leslie M, Tarrant C, et al. Explaining Matching Michigan: an ethnographic study of a patient safety program. *Implement Sci*. 2013;8:70.

7. Zohar D. Safety climate in industrial organizations: Theoretical and applied implications. *J Appl Psychol*. 1980;65:96–102.

8. Fan CJ, Pawlik TM, Daniels T, et al. Association of Safety Culture with Surgical Site Infection Outcomes. *J Am Coll Surg*. 2016;222:122–128.

9. Lin DM, Carson KA, Lubomski LH, et al. Statewide Collaborative to Reduce Surgical Site Infections : Results of the Hawaii Surgical Unit-Based Safety Program. *J Am Coll Surg*. 2018;227:189-197.e1.

10. Atkinson A, Eisenring M-C, Troillet N, et al. Surveillance quality correlates with surgical site infection rates in knee and hip arthroplasty and colorectal surgeries: A call to action to adjust reporting of SSI rates. *Infect Control Hosp Epidemiol*. 2021;42:1451–1457.

11. Zohar Dov, Luria G. A Mulitlevel Model of Safety Climate: Cross-Level Relationships Between Organization an Group-Level Climates. *J Appl Psychol*. 2005;90:616–628.

12. Ginsburg L, Gilin Oore D. Patient safety climate strength: a concept that requires more attention. *BMJ Qual Saf*. 2016;25:680–687.

For peer review only - http://bmjopen.bmj.com/site/about/guidelines.xhtml

13. Weaver SJ, Weeks K, Pham JC, et al. On the CUSP: Stop BSI: Evaluating the relationship between central line-associated bloodstream infection rate and patient safety climate profile. *Am J Infect Control*. 2014;42:S203–S208.

14. Mascherek AC, Schwappach DLB. Patient safety climate profiles across time: Strength and level of safety climate associated with a quality improvement program in Switzerland: A cross-sectional survey study. *PLoS ONE*. 2017;12:e0181410.

15. Tawfik DS, Thomas EJ, Vogus TJ, et al. Safety climate, safety climate strength, and length of stay in the NICU. *BMC Health Serv Res*. 2019;19:738.

16. Profit J, Sharek PJ, Kan P, et al. Teamwork in the NICU Setting and Its Association with Health Care – Associated Infections in Very Low-Birth-Weight Infants. *Am J Perinatol*. 2017;34:1032–1039.

17. Gambashidze N, Hammer A, Wagner A, et al. Influence of Gender, Profession, and Managerial Function on Clinicians' Perceptions of Patient Safety Culture: A Cross-National Cross-Sectional Study. *J Patient Saf*. 2021;17:e280–e287.

18. Singer SJ, Falwell A, Gaba DM, et al. Patient safety climate in US hospitals: variation by management level. *Med Care*. 2008;46:1149–1156.

19. Kristensen S, Hammer A, Bartels P, et al. Quality management and perceptions of teamwork and safety climate in European hospitals. *Int J Qual Health Care*. 2015;27:498–505.

20. Morris TP, White IR, Royston P. Tuning multiple imputation by predictive mean matching and local residual draws. *BMC Med Res Methodol*. 2014;14:75.

21. Sexton JB, Helmreich RL, Neilands TB, et al. The Safety Attitudes Questionnaire: psychometric properties, benchmarking data, and emerging research. *BMC Health Serv Res*. 2006;6:44.

22. Gehring K, Mascherek AC, Bezzola P, et al. Safety climate in Swiss hospital units : Swiss version of the Safety Climate Survey. *J Eval Clin Pract*. 2015;21:332–338.

23. LeBreton JM, Senter JL. Answers to 20 Questions About Interrater Reliability and Interrater Agreement. *Organ Res Methods*. 2008;11:815–852.

For peer review only - http://bmjopen.bmj.com/site/about/guidelines.xhtml

24. Zohar Dov, Luria G. A Mulitlevel Model of Safety Climate: Cross-Level Relationships Between Organization an Group-Level Climates. *J Appl Psychol*. 2005;90:616–628.

25. Schneider B, Salvaggio AN, Subirats M. Climate strength: a new direction for climate research. *J Appl Psychol*. 2002;87:220–229.

26. Culver DH, Horan TC, Gaynes RP, et al. Surgical wound infection rates by wound class, operative procedure, and patient risk index. *Am J Med*. 1991;91:S152–S157.

27. Kuster SP, Eisenring M-C, Sax H, et al. Structure, Process, and Outcome Quality of Surgical Site Infection Surveillance in Switzerland. *Infect Control Hosp Epidemiol*. 2017;38:1172–1181.

28. Weick KE, Sutcliffe KM, Obstfeld D. Organizing for high reliability: Processes of collective mindfulness. In: Research in Organizational Behavior, Vol. 21, 1999. 1999:81–123.

29. Gambashidze N, Hammer A, Wagner A, et al. Influence of Gender, Profession, and Managerial Function on Clinicians' Perceptions of Patient Safety Culture. *J Patient Saf*. 2019;00:1.

30. Singer SJ, Falwell A, Gaba DM, et al. Patient safety climate in US hospitals: variation by management level. *Med Care*. 2008;46:1149–1156.

31. Makary MA, Sexton JB, Freischlag JA, et al. Operating Room Teamwork among Physicians and Nurses: Teamwork in the Eye of the Beholder. *J Am Coll Surg*. 2006;202:746–752.

32. Okuyama JHH, Galvao TF, Silva MT. Healthcare Professional's Perception of Patient Safety Measured by the Hospital Survey on Patient Safety Culture: A Systematic Review and Meta-Analysis. *Sci. World J*. 2018;2018:1–11.

33. Fan CJ, Pawlik TM, Daniels T, et al. Association of Safety Culture with Surgical Site Infection Outcomes. *J Am Coll Surg*. 2016;222:122–128.

34. Lee SE, Scott LD, Dahinten VS, et al. Safety Culture, Patient Safety, and Quality of Care Outcomes: A Literature Review. *West J Nurs Res*. 2019;41:279–304.

35. DiCuccio MH. The Relationship Between Patient Safety Culture and Patient Outcomes. *J Patient Saf*. 2015;11:135–142.

For peer review only

For peer review only - http://bmjopen.bmj.com/site/about/guidelines.xhtml

36. Hofmann DA, Mark B. An investigation of the relationships between safety climate and medication errors as well as other nurse and patient outcomes. *Personnel Psychology*.;59:847–869.

37. Groves PS. The Relationship Between Safety Culture and Patient Outcomes: Results From Pilot Meta-Analyses. *West J Nurs Res*. 2014;36:66–83.

38. Churruca K, Ellis LA, Pomare C, et al. Dimensions of safety culture: a systematic review of quantitative, qualitative and mixed methods for assessing safety culture in hospitals. *BMJ Open*. 2021;11:43982.

For peer review only

For peer review only - http://bmjopen.bmj.com/site/about/guidelines.xhtml

Figures

Figure 1: Scores plotted against NHSN adjusted infection rate

Bubble sizes proportional to standard error of the infection rate; weighted linear model shown (red solid) with 95% confidence interval (grey shaded); LOESS smoother added (blue dashed); weights are proportional to number of participants answering the safety climate survey in the respective hospital.

A. Hip and knee arthroplasty

i. Safety climate ii. Teamwork

NHSN: National Healthcare Safety Network

B. Colorectal surgery

i. Safety climate ii. Teamwork

C. Cesarean section

i. Safety climate ii. Teamwork

For peer review only - http://bmjopen.bmj.com/site/about/guidelines.xhtml

Figure 2: Standard deviation of score plotted against NHSN adjusted infection rate

A. Hip and knee arthroplasty

i. Safety climate strength

ii. Teamwork climate strength

B. Colorectal surgery

i. Safety climate strength

ii. Teamwork climate strength

C. Cesarean section

i. Safety climate strength

ii. Teamwork climate strength

Figure 3: Differences in SSI rate and safety climate according to hospital size

Figure 4: Differences in safety climate ratings according to SSI surveillance quality, stratified by hospital size

For peer review only

For peer review only - http://bmjopen.bmj.com/site/about/guidelines.xhtml

ACKNOWLEDGMENTS

The authors thank all nurses, physicians who responded to the survey and to the local coordinators facilitating the survey in the hospitals. We also thank Maria Mancuso (Clinica Luganese Moncucco) as well as Adriana Degiorgi and Céline Corti (both Ente Ospedaliero Cantonale) for facilitating the pre-test of the Italian survey items.

Members of the Watussi Study Group (in alphabetical order): Andrew Atkinson, PhD (Inselspital, Bern University Hospital and University of Bern), Lauro Damonti, MD (Inselspital, Bern University Hospital and University of Bern), Philipp Jent, MD (Inselspital, Bern University Hospital and University of Bern), Rüdiger Külpmann, PhD (Lucerne University of Applied Sciences and Arts), Judith Maag, MPH (Inselspital, Bern University Hospital and University of Bern), Jonas Marschall, MD (Inselspital, Bern University Hospital and University of Bern), Yvonne Pfeiffer, Dr. sc. (Swiss Patient Safety Foundation), Vanja Piezzi, MD (Inselspital, Bern University Hospital and University of Bern), Luisa Salazar, PhD (Inselspital, Bern University Hospital and University of Bern), David Schwappach, PhD (Swiss Patient Safety Foundation), Benoit Sicre, PhD (Lucerne University of Applied Sciences and Arts), Bernard Surial, MD (Inselspital, Bern University Hospital and University of Bern), Nicolas Troillet, MD (Central Institute of Valais Hospitals), Andreas Widmer, MD (University Hospital Basel), Marcel Zwahlen, PhD (Institute of Social and Preventive Medicine, University of Bern).

For peer review only

For peer review only - http://bmjopen.bmj.com/site/about/guidelines.xhtml

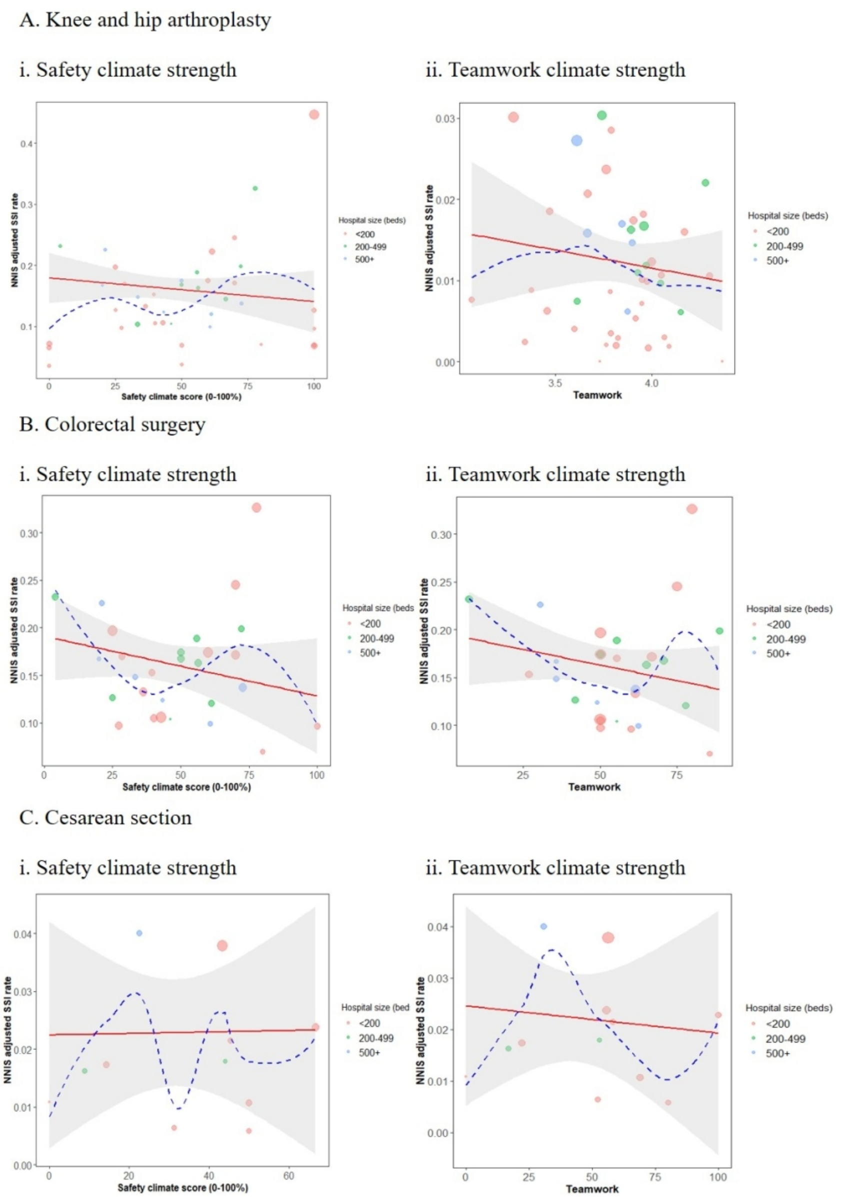

Figure 1: Scores plotted against NHSN adjusted infection rate

191x271mm (150 x 150 DPI)

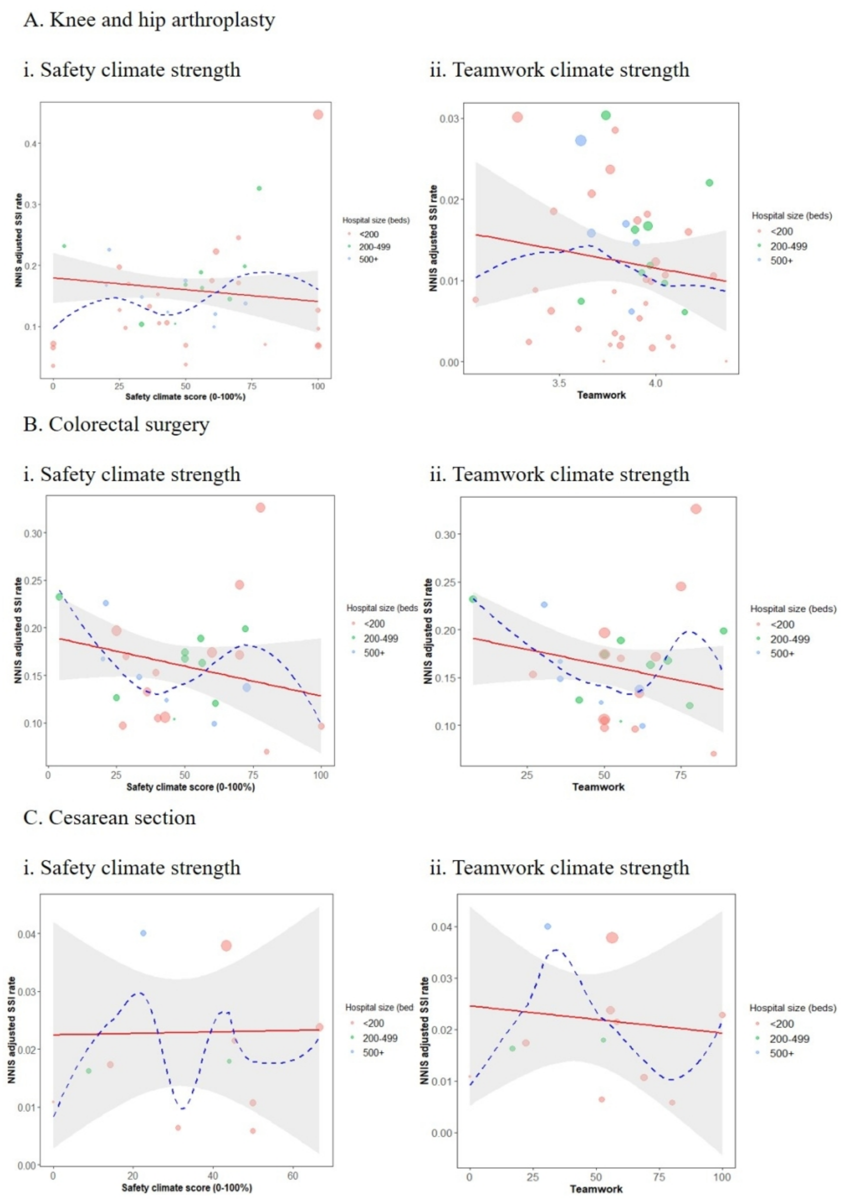

Figure 2: Standard deviation of score plotted against NHSN adjusted infection rate

191x271mm (150 x 150 DPI)

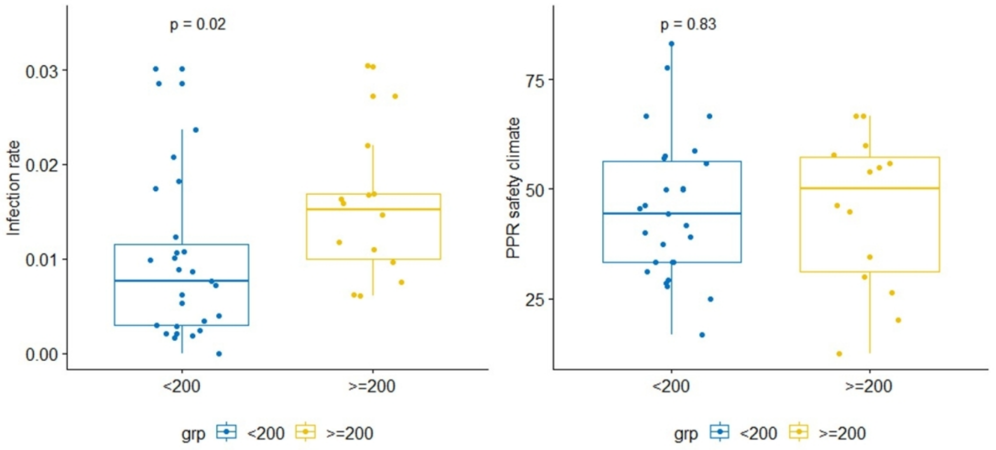

Figure 3: Differences in SSI rate and safety climate according to hospital size

250x116mm (150 x 150 DPI)

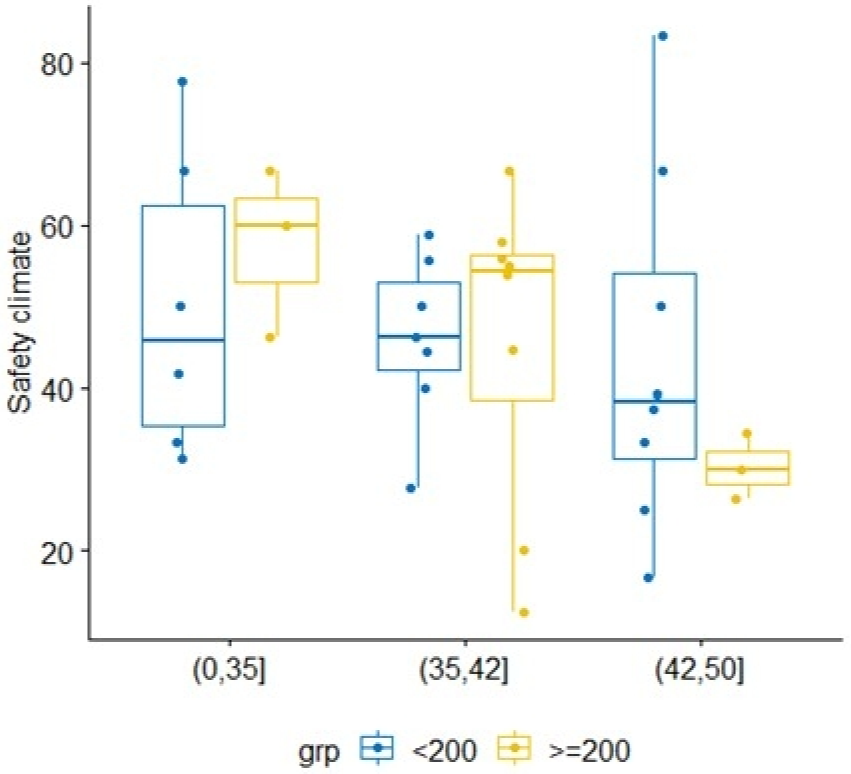

Figure 4: Differences in safety climate ratings according to SSI surveillance quality, strati-fied by hospital size

114x106mm (96 x 96 DPI)

Supplementary Tables

Table S1: Patient characteristics for all hip and knee arthroplasties, colorectal, and Cesarean section procedures in 2017-19

**Characteristic**<br>N (%) / median [IQR]	**Hip and knee**	**Colorectal**	**Cesarean section**
Number of surgeries	58’495	21’445	19’917
Number of hospitals	127	126	49
Surgical site infection	564 (1.0%)	2’973 (13.9%)	405 (2.0%)
Hospital size (beds)			
<200	42’200 (72.1%)	8’625 (40.2%)	13’991 (70.2%)
200-499	13’640 (23.3%)	8’494 (39.6%)	5’137 (25.8%)
500+	2’655 (4.5%)	4’326 (20.2%)	789 (4.0%)
Procedure Type			
Knee	24’711 (42.2%)		
Rectal		888 (4.1%)	
Class			
I (clean)	58’495 (100.0%)	0 (0.0%)	0 (0.0%)
II (potentially contaminated)	-	14’835 (69.2%)	14’281 (71.7%)
III (contaminated)	-	2’548 (11.9%)	5’235 (26.3%)
IV (infected)	-	4’062 (18.9%)	401 (2.0%)
Duration of procedure (mins)	80 [63, 102]	165 [120, 223]	40 [31, 51]
Age (years)	70 [61, 76]	69 [58, 77]	33 [30, 37]
Sex – female	32’226 (55.1%)	10’849 (50.6%)	19’917 (100.0%)
Overlong operation (*ScoreT*)	7’187 (12.3%)	9’025 (42.1%)	2’537 (12.7%)
ASA levels 3/4/5	16’777 (28.7%)	9’701 (45.2%)	1’095 (5.5%)
Minimal invasive	24’071 (41.2%)		
Endoscopic		12’261 (57.2%)	
Elective surgery	58’355 (99.8%)	15’532 (72.4%)	9’636 (48.4%)
Year			
2017	21’297 (36.4%)	6’902 (32.2%)	7’106 (35.7%)
2018	20’926 (35.8%)	7’350 (34.3%)	6’528 (32.8%)
2019	16’272 (27.8%)	7’193 (33.5%)	6’283 (31.5%)

IQR Inter Quartile Range; ASA American Society of Anesthesiology

For peer review only

Table S2: Patient characteristics of those included in the analysis compared to those from all surgeries

A. Hip and knee arthroplasty

**Characteristic**<br>N (%) / median [IQR]	**Analysis set**	**All surgeries**
Number of surgeries	20’434	58’495
Number of hospitals	41	127
Surgical site infection	205 (1.0%)	564 (1.0%)
Hospital size (beds)		
<200	15960 (78.1%)	45’475 (77.7%)
200-499	2940 (14.4%)	11’504 (18.9%)
500+	1534 (7.5%)	1’966 (3.4%)
Procedure Type		
Knee	6727 (32.9%)	24’711 (42.2%)
Class		
I (clean)	20434 (100.0%)	58’495 (100.0%)
II (potentially contaminated)	-	-
III (contaminated)	-	-
IV (infected)	-	-
Duration of procedure (mins)	76 [59, 97]	80 [63, 102]
Age (years)	70 [61, 76]	70 [61, 76]
Sex – female	10933 (53.5%)	32’226 (55.1%)
Overlong operation (*ScoreT*)	2096 (10.3%)	7’187 (12.3%)
ASA levels 3/4/5	5957 (29.2%)	16’777 (28.7%)
Minimal invasive	10123 (49.5)	24’071 (41.2%)
Elective surgery	20388 (99.8%)	58’355 (99.8%)
Year		
2017	7212 (35.3%)	21’297 (36.4%)
2018	7288 (35.7%)	20’926 (35.8%)
2019	5934 (29.0%)	16’272 (27.8%)

For peer review only

For peer review only - http://bmjopen.bmj.com/site/about/guidelines.xhtml

B. Colorectal procedures

**Characteristic**<br>N (%) / median [IQR]	**Analysis set**	**All surgeries**
Number of surgeries	8’321	21’445
Number of hospitals	28	126
Surgical site infection	1’301 (15.6%)	2’973 (13.9%)
Hospital size (beds)		
<200	2’442 (29.3%)	8’622 (40.2%)
200-499	3’115 (37.4%)	7’970 (37.2%)
500+	2’764 (33.2%)	4’853 (22.6%)
Procedure Type		
Rectal	396 (4.8%)	888 (4.1%)
Class		
I (clean)	0 (0.0%)	0 (0.0%)
II (potentially contaminated)	5’641 (67.8%)	14’835 (69.2%)
III (contaminated)	995 (12.0%)	2’548 (11.9%)
IV (infected)	1’685 (20.2%)	4’062 (18.9%)
Duration of procedure (mins)	155 [115, 213]	165 [120, 223]
Age (years)	69 [57, 77]	69 [58, 77]
Sex – female	4’076 (49.0%)	10’849 (50.6%)
Overlong operation (*ScoreT*)	3’131 (37.6%)	9’025 (42.1%)
ASA levels 3/4/5	3’982 (47.9%)	9’701 (45.2%)
Endoscopic	4556 (54.8%)	12’261 (57.2%)
Elective surgery	5831 (70.1%)	15’532 (72.4%)
Year		
2017	2’621 (31.5%)	6’902 (32.2%)
2018	2’799 (33.6%)	7’350 (34.3%)
2019	2’901 (34.9%)	7’193 (33.5%)

For peer review only

C. Cesarean sections

**Characteristic**<br>N (%) / median [IQR]	**Analysis set**	**All surgeries**
Number of surgeries	4’346	19’917
Number of hospitals	11	49
Surgical site infection	89 (2.0%)	405 (2.0%)
Hospital size (beds)		
<200	2429 (55.9%)	13’991 (70.2%)
200-499	1128 (26.0%)	5’137 (25.8%)
500+	789 (18.2%)	789 (4.0%)
Class		
I (clean)	0 (0.0%)	0 (0.0%)
II (potentially contaminated)	3313 (76.2%)	14’281 (71.7%)
III (contaminated)	993 (22.8%)	5’235 (26.3%)
IV (infected)	40 (0.9%)	401 (2.0%)
Duration of procedure (mins)	42 [33, 52]	40 [31, 51]
Age (years)	33 [30,36]	33 [30, 37]
Overlong operation (*ScoreT*)	563 (13.0%)	2’537 (12.7%)
ASA levels 3/4/5	244 (5.6%)	1’095 (5.5%)
Elective surgery	2’119 (48.8%)	9’636 (48.4%)
Year		
2017	1276 (29.4%)	7’106 (35.7%)
2018	1’520 (35.0%)	6’528 (32.8%)
2019	1550 (35.7%)	6’283 (31.5%)

For peer review only

Patient level analysis

Table S3: Uni- and multivariable estimates from the fitted logistic regression models

A. Hip and knee arthroplasty

**Endpoint:**	Univariable		Multivariable	
**SSI (0/1)**	OR (95% CI)	p-value	OR (95% CI)	p-value
Sex - female	0.8 [0.6, 1.0]	0.05	NS	
Age (in 10 year steps)		NS	-	
ASA – levels 3/4/5	2.2 [1.6, 3.0]	<0.001	2.2 [1.5, 3.0]	<0.001
Overlong operation ("scoreT")	1.8 [1.1, 3.0]	0.02	1.8 [1.1, 2.9]	0.03
Class		nE	-	
Minimally invasive		NS		
Operation type - Knee (reference Hip)		NS		
Hospital size (number of beds)				
<200	1 (reference)			
200-500	1.5 [0.8, 2.8]	NS		
500+	2.0 [1.0, 4.0]	0.05	NS	
Safety climate (per hospital)		NS	-	
Teamwork (per hospital)		NS	-	
% managerial role (per hospital)		NS	-	
% physician (per hospital)		NS	-	

NS: not significant at the 5% level; nE: not estimated

For peer review only - http://bmjopen.bmj.com/site/about/guidelines.xhtml

B. Colorectal procedures

**Endpoint:**	Univariable		Multivariable	
**SSI (0/1)**	OR (95% CI)	p-value	OR (95% CI)	p-value
Sex - female	0.8 [0.7, 0.9]	0.005	0.8 [0.7, 1.0]	0.03
Age (in 10 year steps)		NS	-	
ASA – levels 3/4/5	1.7 [1.4, 2.0]	<0.001	1.3 [1.1, 1.6]	0.002
Overlong operation (“scoreT”)	1.5 [1.3, 1.8]	<0.001	1.4 [1.2, 1.7]	<0.001
Class				
II clean-contaminated	1 (reference)			
III contaminated	1.0 [0.8, 1.3]	NS	NS	
IV infected	1.7 [1.4, 2.0]	<0.001	1.3 [1.1, 1.6]	0.01
Endoscopic	0.5 [0.4, 0.6]	<0.001	0.5 [0.4, 0.6]	<0.001
Operation type - rectal	1.3 [1.0, 1.7]	0.07	1.3 [1.0, 1.7]	0.05
Hospital size (number of beds)		NS		
<200				
200-500				
500+				
Safety climate (per hospital)		NS		
Teamwork (per hospital)		NS		
% managerial role (per hospital)	1.0 [1.0, 1.0]	0.1		NS
% physician (per hospital)		NS		

For peer review only

For peer review only - http://bmjopen.bmj.com/site/about/guidelines.xhtml

B. Cesarean sections

**Endpoint:**	Univariable		Multivariable	
**SSI (0/1)**	OR (95% CI)	p-value	OR (95% CI)	p-value
Age (in 10 year steps)	0.7 [0.5, 1.0]	0.05		NS
ASA – levels 3/4/5		NS		
Overlong operation (“scoreT”)		NS		
Class				
II clean-contaminated	1 (reference)		1 (reference)	
III contaminated	NS		NS	
IV infected	4.4 [2.3, 8.6]	0.002	3.5 [1.7, 7.2]	0.02
Hospital size (number of beds)				
<200	1 (reference)		1 (reference)	
200-500	NS		NS	
500+	2.7 [1.9, 3.9]	<0.001	2.8 [2.0, 4.0]	0.001
Safety climate (per hospital)		NS		
Teamwork (per hospital)		NS		
% managerial role (per hospital)		NS		
% physician (per hospital)	1.0 [1.0, 1.0]	0.02		NS

For peer review only

For peer review only - http://bmjopen.bmj.com/site/about/guidelines.xhtml

Table M1 Descriptive statistics of complete cases and following multiple imputation for knee and hip surgeries

	Hip and knee		
median [IQR]	Complete cases	Multiply imputed (K=20)	p-value
Safety climate (%)	45.5 [12.5, 83.3]	50 [16, 87]	0.09
% leaders	20% [0, 50]	25% [6, 55]	<0.001
% physician	30% [0, 100]	44% [0, 100]	<0.001
Teamwork (%)	50% [14, 89]	55 [10, 92]	0.19
% leaders	20% [0, 50]	25% [6, 55%]	<0.001
% physician	33% [0, 100]	44% [0, 100]	<0.001

Table M2: Comparison of estimates from the fitted weighted linear model for knee and hip surgeries

	Hip and knee<br>slope estimate (95% CI), p-value	
Endpoint	Complete cases	Multiply imputed (K=20)
% safety climate regressed on infection rate1	-100 (-215, 15), p=0.10	-182 (-296, -68), p=0.003
% Teamwork regressed on infection rate1	-59 (-186, 67.7), p=0.37	-98 (-218, 22), p=0.11

[1]square root of the infection rate

For peer review only - http://bmjopen.bmj.com/site/about/guidelines.xhtml

Supplementary Material

Figure S1: Patient cascade and selection for hip and knee , colorectal and cesarean section procedures for the safety climate endpoint (complete case analysis).

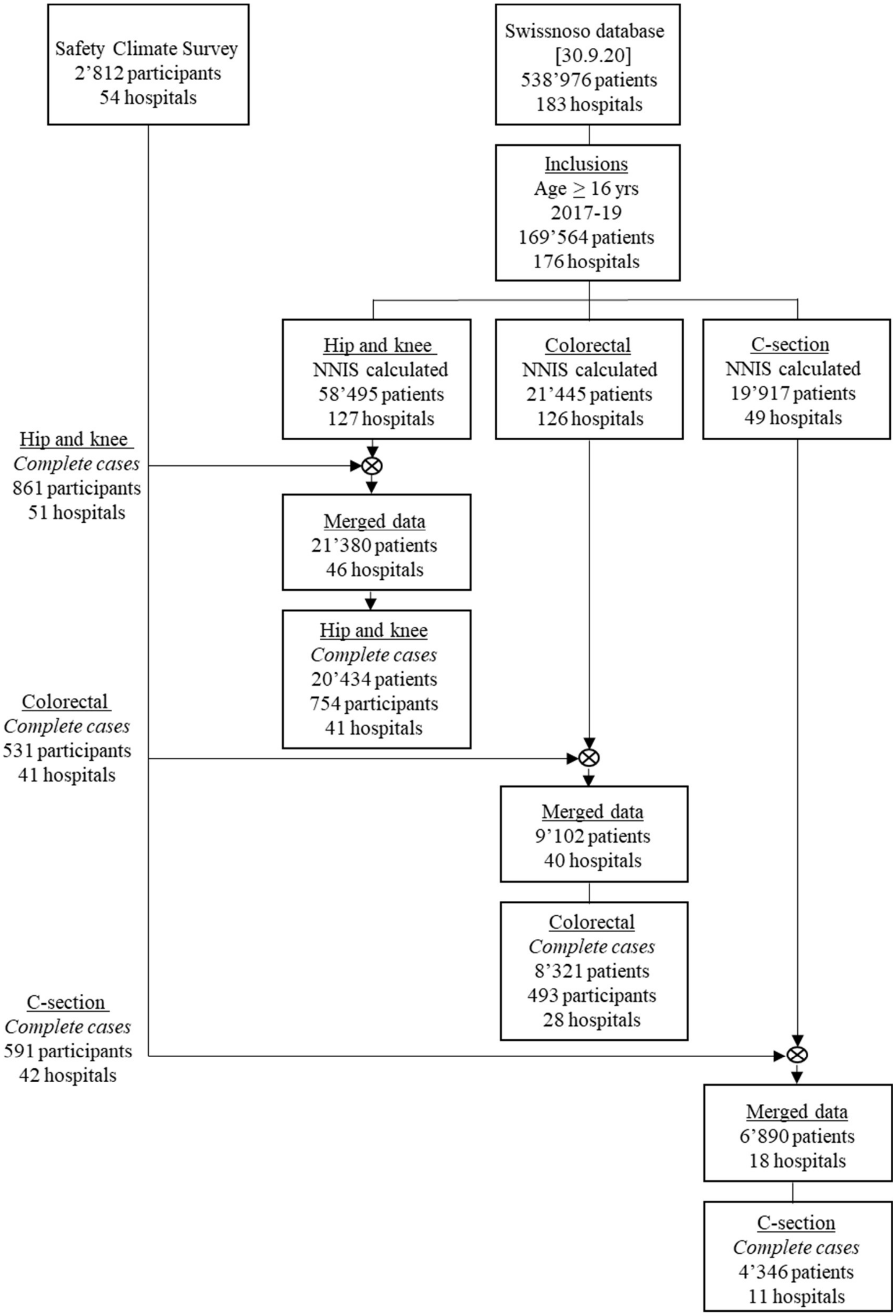

Figure S2A: Boxplot of calculated NNIS adjusted SSI rates per hospital, stratified by surgery type

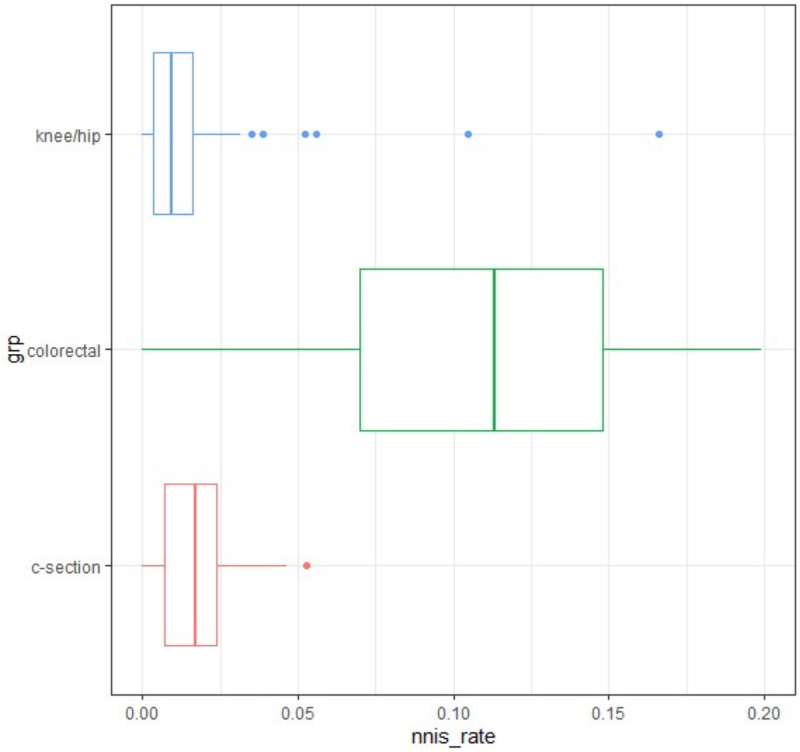

For peer review only - http://bmjopen.bmj.com/site/about/guidelines.xhtml

eview only

Figure S2B: Boxplots of calculated percentage safety climate (left) and teamwork (right) per hospital, stratified by surgery type for the complete cases from the participants

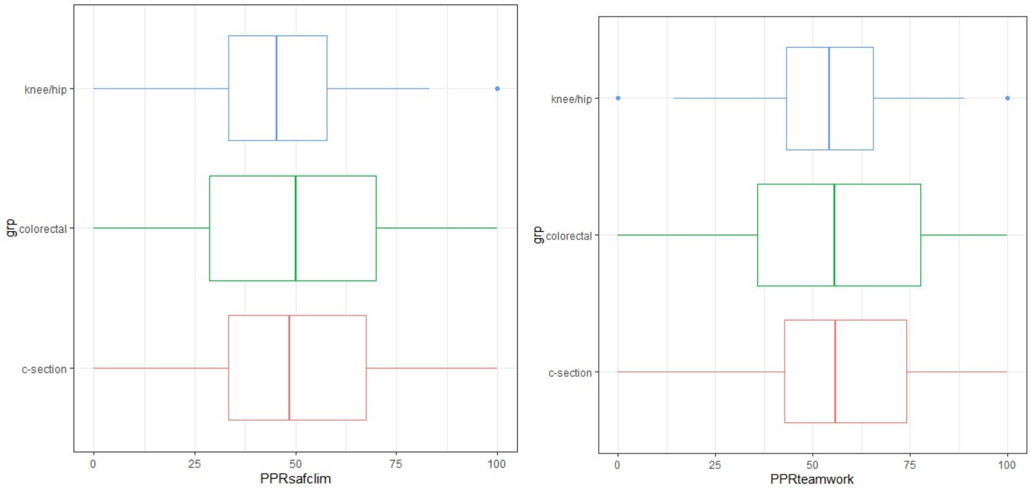

peer review only

For peer review only - http://bmjopen.bmj.com/site/about/guidelines.xhtml

Figure S3: Safety climate (A) and teamwork (B), stratified by hospital size

<u>Hip and knee arthroplasty</u>

A: Safety climate and hospital size

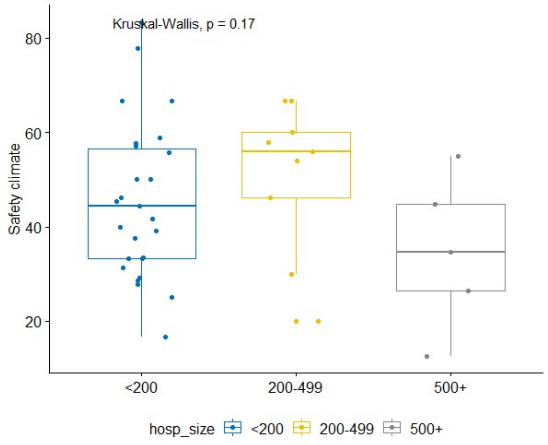

B. Teamwork and hospital size

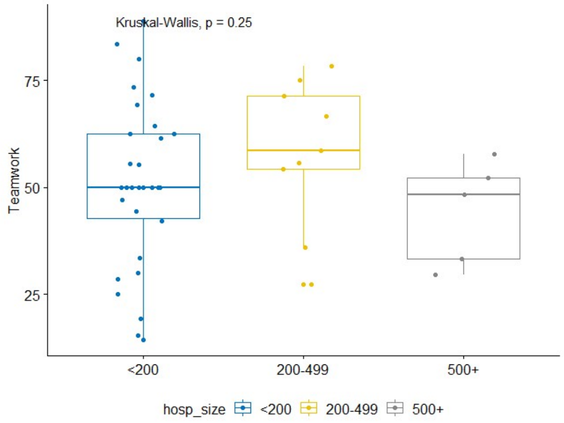

C. Infection and hospital size

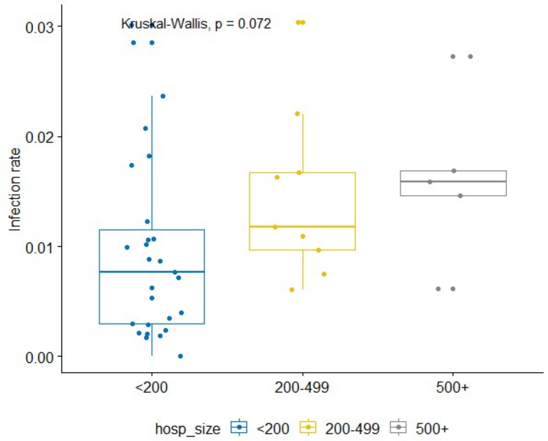

For peer review only - http://bmjopen.bmj.com/site/about/guidelines.xhtml

Colorectal

A: Safety climate and hospital size

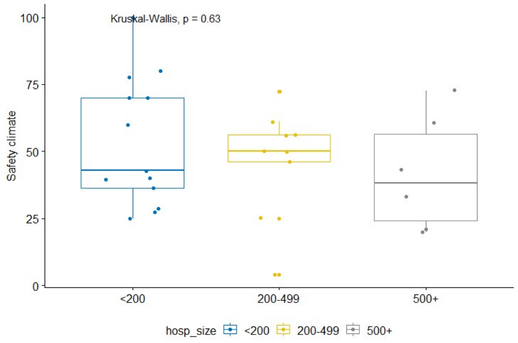

B. Teamwork and hospital size

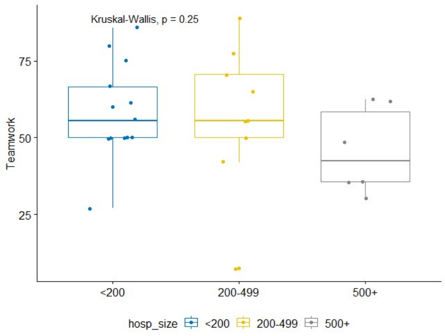

C. Infection and hospital size

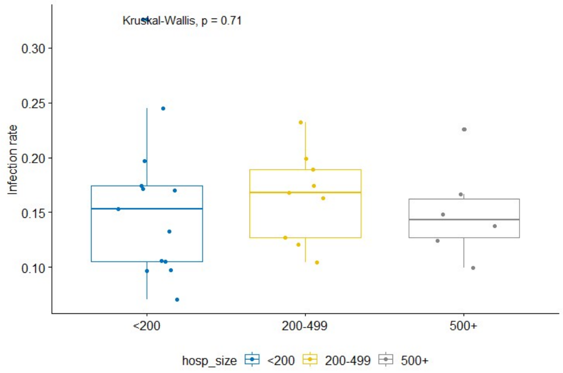

For peer review only - http://bmjopen.bmj.com/site/about/guidelines.xhtml

Cesarean section

A: Safety climate and hospital size

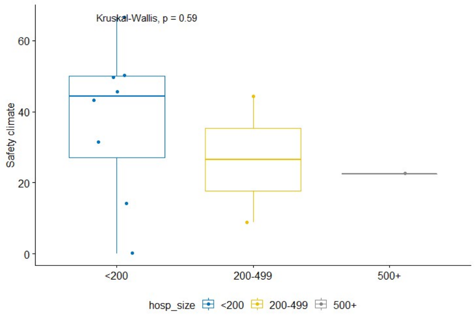

B. Teamwork and hospital size

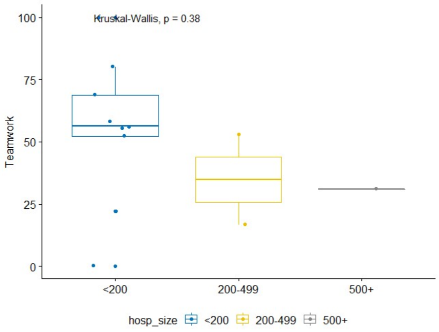

C. Infection and hospital size

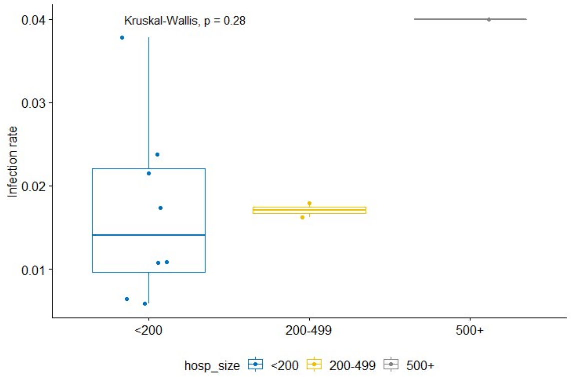

For peer review only - http://bmjopen.bmj.com/site/about/guidelines.xhtml

APPENDIX

	Recoded
**SCS Safety Climate Scale (SCS)**	
(1) The culture of this clinical area makes it easy to learn from the mistakes of others.	
(2) Medical errors** are handled appropriately in this clinical area. ** Medical error is defined as any mistake in the delivery of care, by any healthcare professional, regardless of outcome.	
(3) The senior leaders in my hospital listen to me and care about my concerns.	
(4) The physician and nurse leaders in my area listen to me and care about my concerns.	
(5) I am encouraged by my colleagues to report any safety concerns I may have.	
(6) I know the proper channels to direct questions regarding patient safety.	
(7) Leadership is driving us to be a safety- centered institution.	
(8) I receive appropriate feedback about my performance.	
(9) I would feel safe being treated here as a patient.	
(10) I am satisfied with the availability of clinical leadership (please respond to all three):	
a) Physician;	
b) Nursing;	
c) Pharmacy	
(11) Briefing personnel before the start of a shift (i.e., to plan for possible contingencies) is an important part of patient safety.	
(12) Briefings are common here.	
(13) This institution is doing more for patient safety now than it did one year ago.	
(14) I believe that most adverse events occur as a result of multiple system failures and are not attributable to one individual's actions.	
(15) The personnel in this clinical area take responsibility for patient safety.	
(16r) Management/leadership does not knowingly compromise safety concerns for productivity.	recoded
(17r) Personnel frequently disregard rules or guidelines that are established for this clinical area.	recoded
(18) Patient safety is constantly reinforced as the priority in this clinical area.	
(19r) In this clinical area, it is difficult to discuss errors.	recoded
(20) My suggestions about safety would be acted upon if I expressed them to management.	
**SCS Safety Climate Scale (SCS)**	
(1) Nurse input is well received in this clinical area.	
(2) In this clinical area, it is difficult to speak up if I perceive a problem with patient care.	recoded
(3) Disagreements in this clinical area are resolved appropriately (i.e., not who is right, but what is best for the patient).	
(4) I have the support I need from other personnel to care for patients.	
(5) It is easy for personnel here to ask questions when there is something that they do not understand.	
(6) The physicians and nurses here work together as a well-coordinated team.	

For peer review only

STROBE Statement—Checklist of items that should be included in reports of ***cross-sectional studies***

	Item No	Recommendation	Page No
**Title and abstract**	1	(*a*) Indicate the study's design with a commonly used term in the title or the abstract	1+3
		(*b*) Provide in the abstract an informative and balanced summary of what was done and what was found	3+4
**Introduction**			
Background/rationale	2	Explain the scientific background and rationale for the investigation being reported	5+6
Objectives	3	State specific objectives, including any prespecified hypotheses	6
**Methods**			
Study design	4	Present key elements of study design early in the paper	6
Setting	5	Describe the setting, locations, and relevant dates, including periods of recruitment, exposure, follow-up, and data collection	7
Participants	6	(*a*) Give the eligibility criteria, and the sources and methods of selection of participants	7,8,9
Variables	7	Clearly define all outcomes, exposures, predictors, potential confounders, and effect modifiers. Give diagnostic criteria, if applicable	8+9
Data sources/ measurement	8*	For each variable of interest, give sources of data and details of methods of assessment (measurement). Describe comparability of assessment methods if there is more than one group	*7,8,9*
Bias	9	Describe any efforts to address potential sources of bias	10, 13, 14
Study size	10	Explain how the study size was arrived at	11
Quantitative variables	11	Explain how quantitative variables were handled in the analyses. If applicable, describe which groupings were chosen and why	7,8,9
Statistical methods	12	(*a*) Describe all statistical methods, including those used to control for confounding	9
		(*b*) Describe any methods used to examine subgroups and interactions	9, 11
		(*c*) Explain how missing data were addressed	11
		(*d*) If applicable, describe analytical methods taking account of sampling strategy	
		(*e*) Describe any sensitivity analyses	
**Results**			
Participants	13*	(a) Report numbers of individuals at each stage of study—eg numbers potentially eligible, examined for eligibility, confirmed eligible, included in the study, completing follow-up, and analysed	25, 29
		(b) Give reasons for non-participation at each stage	29, 7-9
		(c) Consider use of a flow diagram	29
Descriptive data	14*	(a) Give characteristics of study participants (eg demographic, clinical, social) and information on exposures and potential confounders	35-38, 42
		(b) Indicate number of participants with missing data for each variable of interest	35-38
Outcome data	15*	Report numbers of outcome events or summary measures	8+9

For peer review only - http://bmjopen.bmj.com/site/about/guidelines.xhtml

Main results	16	(*a*) Give unadjusted estimates and, if applicable, confounder-adjusted estimates and their precision (eg, 95% confidence interval). Make clear which confounders were adjusted for and why they were included	10, 11
		(*b*) Report category boundaries when continuous variables were categorized	10, 11
		(*c*) If relevant, consider translating estimates of relative risk into absolute risk for a meaningful time period	
Other analyses	17	Report other analyses done—eg analyses of subgroups and interactions, and sensitivity analyses	11
**Discussion**			
Key results	18	Summarise key results with reference to study objectives	11+12
Limitations	19	Discuss limitations of the study, taking into account sources of potential bias or imprecision. Discuss both direction and magnitude of any potential bias	13+14
Interpretation	20	Give a cautious overall interpretation of results considering objectives, limitations, multiplicity of analyses, results from similar studies, and other relevant evidence	12-15
Generalisability	21	Discuss the generalisability (external validity) of the study results	13-15
**Other information**			
Funding	22	Give the source of funding and the role of the funders for the present study and, if applicable, for the original study on which the present article is based	2

*Give information separately for exposed and unexposed groups.

Note: An Explanation and Elaboration article discusses each checklist item and gives methodological background and published examples of transparent reporting. The STROBE checklist is best used in conjunction with this article (freely available on the Web sites of PLoS Medicine at http://www.plosmedicine.org/, Annals of Internal Medicine at http://www.annals.org/, and Epidemiology at http://www.epidem.com/). Information on the STROBE Initiative is available at www.strobe-statement.org.

For peer review only

BMJ Open

Are cross-sectional safety climate survey results in operation room staff associated with the surgical site infection rates in Swiss hospitals?

Journal:	*BMJ Open*
Manuscript ID	bmjopen-2022-066514.R1
Article Type:	Original research
Date Submitted by the Author:	16-Dec-2022
Complete List of Authors:	Pfeiffer, Yvonne; Stiftung fur Patientensicherheit<br>Atkinson, Andrew; Inselspital University Hospital Bern, Department of Infectious Diseases<br>Maag, Judith; Inselspital University Hospital Bern, Department of Infectious Diseases; Swissnoso, National Center for Infection Control, Bern, Switzerland<br>Lane, Michael; Parkland Health and Hospital System<br>Schwappach, David; Universität Bern, Institute of Social and Preventive Medicine<br>Marschall, Jonas; Inselspital Universitatsspital Bern, Department of Infectious Diseases; Washington University School of Medicine in Saint Louis, Division of Infectious Diseases
**Primary Subject Heading**:	Health services research
Secondary Subject Heading:	Surgery, Infectious diseases
Keywords:	Health & safety < HEALTH SERVICES ADMINISTRATION & MANAGEMENT, Risk management < HEALTH SERVICES ADMINISTRATION & MANAGEMENT, Infection control < INFECTIOUS DISEASES, PUBLIC HEALTH, Adult surgery < SURGERY

SCHOLARONE™
Manuscripts

I, the Submitting Author has the right to grant and does grant on behalf of all authors of the Work (as defined in the below author licence), an exclusive licence and/or a non-exclusive licence for contributions from authors who are: i) UK Crown employees; ii) where BMJ has agreed a CC-BY licence shall apply, and/or iii) in accordance with the terms applicable for US Federal Government officers or employees acting as part of their official duties; on a worldwide, perpetual, irrevocable, royalty-free basis to BMJ Publishing Group Ltd ("BMJ") its licensees and where the relevant Journal is co-owned by BMJ to the co-owners of the Journal, to publish the Work in this journal and any other BMJ products and to exploit all rights, as set out in our licence.

The Submitting Author accepts and understands that any supply made under these terms is made by BMJ to the Submitting Author unless you are acting as an employee on behalf of your employer or a postgraduate student of an affiliated institution which is paying any applicable article publishing charge ("APC") for Open Access articles. Where the Submitting Author wishes to make the Work available on an Open Access basis (and intends to pay the relevant APC), the terms of reuse of such Open Access shall be governed by a Creative Commons licence – details of these licences and which Creative Commons licence will apply to this Work are set out in our licence referred to above.

Other than as permitted in any relevant BMJ Author's Self Archiving Policies, I confirm this Work has not been accepted for publication elsewhere, is not being considered for publication elsewhere and does not duplicate material already published. I confirm all authors consent to publication of this Work and authorise the granting of this licence.

For peer review only

Title

Are cross-sectional safety climate survey results in operation room staff associated with the surgical site infection rates in Swiss hospitals?

Brief Title

SSI rate association with safety climate

Authors

Yvonne Pfeiffer*[1], Dr. sc.; Andrew Atkinson[2], PhD; Judith Maag[2,3] MSc., M.A., MPH; Michael A Lane, MD, MPHS[5]; David L. B. Schwappach[4 +] PhD, MPH.; Jonas Marschall[2,3,6 +], MD, and Swissnoso[3].

[1]Swiss Patient Safety Foundation, Zurich, Switzerland; Asylstr. 77, 8032 Zurich, Switzerland, Tel. 0041 43 2441480, Fax 0041 43 2441481, E-mail: ypfeifferdrsc@gmail.com

[2]Department of Infectious Diseases, Bern University Hospital, Inselspital, University of Bern, Bern, Switzerland.

[3]Swissnoso, National Center for Infection Control, Bern, Switzerland

[4]Institute of Social and Preventive Medicine (ISPM), University of Bern, Switzerland

[5]Parkland Health, Dallas, Texas, United States

[6] Division of Infectious Diseases, Washington University School of Medicine, Saint Louis, MO, United States

[+]These authors contributed equally

Corresponding author: Yvonne Pfeiffer, Swiss Patient Safety Foundation, Zurich, Switzerland; Asylstr. 77, 8032 Zurich, Switzerland, Tel. 0041 43 2441480, Fax 0041 43 2441481, E-mail: ypfeifferdrsx@gmail.com

WORD COUNT: 3828

For peer review only

STRUCTURED ABSTRACT

Objectives. The aim of this study was to investigate the association between surgical site infections (SSI), a major source of patient harm, and safety and teamwork climate. Prior research has been unclear regarding this relationship.

Design. Based on the swiss national SSI surveillance and a survey study assessing a) safety climate and b) teamwork climate, associations were analyzed for three kinds of surgical procedures.

Setting and participants. SSI surveillance data from 20'434 surgeries for hip and knee arthroplasty from 41 hospitals, 8'321 for colorectal procedures from 28 hospitals and 4'346 cesarean sections from 11 hospitals and survey responses from Swiss operating room personnel (N=2769) in 54 acute care hospitals.

Primary and secondary outcomes. The primary endpoint of the study was the 30 day (all types) or 1 year (knee/hip with implants) NHSN-adjusted SSI rate. Its association with climate level and strength was investigated in regression analyses, accounting for respondents' professional background, managerial role, and hospital size as confounding factors.

Results. Plotting climate *levels* against infection rates revealed a general trend with SSI rate decreasing as the safety climate increased, but none of the associations were significant (5% level). Linear models for hip and knee arthroplasties showed a negative association between SSI rate and climate perception (p=0.02). For climate *strength*, there were no consistent patterns, indicating that alignment of perceptions was not associated with lower infection rates. Being in a managerial role and being a physician (vs. a nurse) had a positive effect on climate levels regarding SSI in hip and knee arthroplasties, whereas larger hospital size had a negative effect.

Conclusions. This study suggests a possible negative correlation between climate level and SSI rate, while for climate strength no associations were found. Future research should study safety climate more specifically related to infection prevention measures to establish clearer links.

For peer review only - http://bmjopen.bmj.com/site/about/guidelines.xhtml

STRENGTHS AND LIMITATIONS OF THIS STUDY

- This study used national surgical site infections surveillance data of high quality, also including infection events after discharge from the hospital.
- For modelling purposes, safety climate and teamwork climate outcomes, as well as level and strength of climate perceptions, were differentiated.
- Including three different types of surgical procedures allowed for controlling for influences stemming from inherent variations in risk profiles depending on the kind of surgery.
- Potential underreporting of surgical site infections may have had a biasing effect on the analysis.
- Aggregating climate on the hospital level was necessary due to the ad hoc formation of surgery teams, but this could conceal certain trends on unit level.

For peer review only

KEYWORDS

Surgical Site Infection; Surgical Site Infection Rate; Safety Climate; Teamwork Climate; Safety Attitude Questionnaire; Operating Room; Operating Room Personnel; Infection Prevention and Control.

For peer review only - http://bmjopen.bmj.com/site/about/guidelines.xhtml

Introduction

Healthcare-associated infections are a major concern for patient safety. Specifically, surgical site infections (SSI) are considered the most common healthcare-associated infection, occurring in 2-5% of inpatient surgeries and represent an economic burden to the healthcare system [1]. SSI rates may be reduced effectively by performing adequate infection prevention measures, such as the use of antibiotic prophylaxis or skin disinfection prior to incision. For example, the adherence to a standardized bundle of prevention measures reduced SSI after colon surgery in a retrospective cohort study, thus improving patient safety [2]. In order for healthcare workers to perform safety-relevant behaviors, safety climate is thought to play an important role. Specifically, compliance with infection prevention measures is thought to be influenced by the prevailing safety climate [3,4], however, only with mixed supporting evidence so far [5]. Similarly, safety culture and climate have been used as an explanatory factor for failed implementation of preventive interventions that were successful in the original studies [6].

"Safety climate" encompasses shared perceptions related to safety policies, procedures, and practices [7] and is expected to guide the safety behavior of workers. There is limited evidence from prior research indicating that safety climate may be linked to healthcare-associated infections, and SSI in particular: Fan et al. [8] found that nine of twelve safety climate dimensions were associated with SSI rates after colon surgery. To evaluate a program reducing SSIs after colorectal surgery in Hawaiian hospitals, Lin et al. [9] assessed safety climate at baseline and after implementation of the program; however, no consistent patterns of association of (change in) SSI rate with (change in) safety climate dimensions could be identified.

In order to carefully evaluate safety climate as a characteristic of a unit or organization [10], climate *strength* needs to be assessed - in addition to the safety climate *level* indicating positive or negative climate evaluations: Climate strength indicates how aligned the individual climate perceptions are with each other in a given unit and is considered a predictor for safety outcomes [11,12] that can provide

For peer review only - http://bmjopen.bmj.com/site/about/guidelines.xhtml

valuable insights. While the level of safety climate scores may not differ or change over time, their strength, indicating consensus or divergence of perceptions regarding safety climate, may do so [13,14].

Based on the broad coverage and longitudinal assessment of SSI captured by the Swiss surveillance system, this study relates SSI rates to safety climate scores. The study aims were to investigate a) whether better safety climate *level* and higher safety climate *strengths* are associated with lower SSI rates, and b) whether these relationships are also true for teamwork climate scores.

A prior study of our group showed that infection rates may vary with hospital size, meaning that larger hospitals have higher rates, confounded by their case mix and type of surgeries investigated [15]. Therefore, hospital size was considered in our analyses. That study also investigated whether quality of surveillance, assessed by external audits, has an influence on the measured SSI rate, and we considered "quality of surveillance" as assessed with the audit score here, too: hospitals conducting a thorough surveillance demonstrate their ability to implement and follow relevant procedures, which we hypothesized may also be related to safety climate. Three types of procedures were included in the study: colorectal surgery, cesarean section, and knee/hip arthroplasties. They were chosen for being the procedures for which the largest number of institutions report SSI rates in Switzerland.

As prior research exploring the relationship between safety climate and SSI rates has advocated [9] and for keeping potential intercorrelations between variables to a minimum, we used one composite safety climate scale. We added the scale 'teamwork climate' because teamwork is important for safe outcomes in surgeries, and it has been linked to healthcare-associated infections in the NICU setting before [16].

Prior research has also shown that those in managerial positions tend to rate safety climate higher than frontline healthcare workers [17–19], and also that the professional background (here "nurse" or "physician") has an influence on safety climate perceptions [17,18]. This has been traced back to various explanations, such as the existence of different management structures, subcultures, or work demands in these two groups. Therefore, in this study, the managerial role and professional background were taken into account when analyzing the relationship with infection rates.

For peer review only - http://bmjopen.bmj.com/site/about/guidelines.xhtml

Methods

Sample and missing data

All hospitals and hospital networks providing acute care and participating in the SSI surveillance module of Swissnoso were invited (N= 143) to participate in the study, of which N=54 institutions agreed. Among these, all operation room (OR) personnel received the survey. An exact response rate cannot be estimated, as total numbers of healthcare workers in operating rooms were not retrievable. However, we know that 38% of all paper surveys sent out were completed. There was a thorough description of the project's aims, that participation was voluntary and the use of the responses on the first page of the survey. Therefore, participation was considered informed consent.

After exclusion of participants with demographic answers that were inconsistent, e.g., indicating that they worked as physician and as nurse at the same time (7 participants), and those with less than 32 (from 52) responses (36 participants), we had a sample of N = 2769 (see figure S1).

For SSI rates, we selected adult (≥16 years) patients with complete follow-up information in 2017-19 (3 years) for elective hip and knee arthroplasties (58'495 procedures, 127 hospitals), colorectal surgeries (21'445 procedures, 126 hospitals), and cesarean sections (19'917 procedures, 49 hospitals) from the Swissnoso database (extraction performed on 30.9.2020). We assumed this would provide an adequate summary of the infection rates at the time of the survey in 2019. Combining survey data with infection rates meant limiting the sample size, as not all surgery types are reported by every hospital. Equally, for us to match survey responses with persons that work in the surveilled surgery types, each survey respondent had to list the type of surgeries they participate in. Data was collected on the patient and participant level, and then aggregated on hospital level. The resulting analysis set consisted of 20'434 surgeries for hip and knee arthroplasty from 41 hospitals, 8'321 for colorectal procedures from 28 hospitals and 4'346 cesarean sections from 11 hospitals (see figure S1 and table S1).

For peer review only - http://bmjopen.bmj.com/site/about/guidelines.xhtml

In addition to the complete case analysis, we multiply imputed missing responses from individual survey participants using predictive mean matching with the 10 nearest neighbors as donor pool in the algorithm [20].

Safety Climate and Teamwork Climate Measures

In order to assess climate, the safety climate (*SCS*, 22 items) and teamwork climate (*TWC*, 6 items) scales of the Safety Attitudes Questionnaire (SAQ)[21] were used (see appendix). The internal consistency of the *SCS* was high: Cronbach Alpha = .89; for *TWC* it was slightly lower with Cronbach Alpha = .78, but still acceptably high.

The survey was sent out in the three national languages: A prior study had developed Swiss versions in German and French [22], and the Italian version was developed by applying a translation and back-translation process using different translators, blinded to the original items. Any differences were resolved, and the items tested in two sites by OR personnel for understandability and correctness. Two different measures were used in the analyses: safety and teamwork climate *level* and safety and teamwork climate *strength*. Climate level was measured as percent positive responses (PPR) per hospital. Percentage of positive respondents was calculated counting each respondent's score as positive for their unit if their mean score on the scale was 4.0 or greater (from a possible score of 1-5), in line with Tawfik et al's approach. [14]. Intra-hospital correlation of the climate level scores (*rwg*, a measure of within-group agreement) was checked in order to justify aggregation on the hospital level: all *rwgs* were above .66, with their median being .78 (*SCS*) and .81 (*TWC*), respectively, indicating moderate to strong agreement [23]. In line with prior research, climate strength was evaluated using the standard deviation of mean climate level scores per hospital [14,24,25].

Assessment and adjustment of SSI rates and incorporation of audit scores

Analyses were conducted separately for the surgery types, because SSI rates are not comparable between specialties since certain procedures are more prone to SSI than others. The primary endpoint of the study was the 30 day (all types) or 1 year (hip/knee with implants) NHSN (National Healthcare

For peer review only - http://bmjopen.bmj.com/site/about/guidelines.xhtml

Safety Network)-adjusted rate of infection [26], and this was assessed in all patients age 16 years and older for the respective hospitals in the 3-year study period. For hip/knee surgeries, only class I (“clean”) surgeries were included, but all classes were included for colorectal surgeries and cesarean sections.

Despite the sophisticated surveillance system employed in Switzerland, the accuracy of the SSI rates may be affected by underreporting. Therefore, audit scores from on-site quality audits were also taken into account. These hospital audit scores indicate how thoroughly the SSI surveillance is conducted in a given hospital, with higher audit scores were linked to higher rates of reported SSI for certain surgeries [15]. Audit scores range from 0 to 50, and cover aspects such as how structured the data assessment is, how well trained the persons conducting it are, among others [27].

Data Analyses

The primary analysis was based on complete cases only and considered the correlation between the SSI rate, aggregated on the hospital level, and the safety climate and teamwork climate.

Categorical variables are shown with the number per category (N) along with the appropriate percentage of the total, with continuous variables summarized as median and inter-quartile range. Group differences were investigated using the Kruskal-Wallis non-parametric test.

We began by visually checking for associations by plotting safety and teamwork climate scores (level and strength) against the standardized infection rates for each procedure type, stratified by hospital size (<200, 200-499, and 500+ beds).

Weighted linear models were fitted with the respective safety climate endpoint as dependent variable, and as independent variables, the SSI rate, percentage of managerial (vs. non-managerial) roles and percentage of physicians (vs. nurses) having responded to the survey in a hospital for the specific surgery type, the hospital size and hospital type (university, regional, or “other”). The number of surgeries in a given hospital was used for the weighting in the models.

For peer review only - http://bmjopen.bmj.com/site/about/guidelines.xhtml

For peer review only

Those variables statistically significant at the 10% level in univariable analyses were included in the multivariable analysis with forwards selection and backwards deletion using the AIC (Akaike information criterion) to determine the most parsimonious model.

Patient and public involvement

To stay safe from SSI is important for patients undergoing surgery, and to study the role of safety climate thus can offer important avenues for improvement. From the time when the project was funded, the public was informed on the website of Swissnoso (National Center for Infection Control, Bern, Switzerland) and on the website and in the newsletter of the patient safety foundation. The patients were not involved in the design and conduct of the study.

Results

Mean safety climate level scores were very similar between the healthcare workers involved in the three procedure types (c-section 43.3, inter-quartile range [0.0, 66.8], hip/knee median PPR 45.5 [12.5, 83.3], colorectal 48.1 [4.0, 100.0]), but as the ranges show, with large variability between hospitals. As expected, standardized infection rates mirrored the underlying population and surgery type differences between the surgery types (hip/knee median 1.0% [0.0, 3.0], colorectal 16.0% [7.0, 33.0], c-section 2.0% [1.0, 4.0], top part of Table 1, supplementary material figure S2). The median number of survey respondents per hospital was deemed adequate, although there were some imbalances due to low numbers in some hospitals. Approximately 20% of responders had a managerial role and approximately 30% were physicians (as opposed to nurses), although the percentage of physicians was somewhat lower for cesarean sections. Interestingly, despite the comparable safety climate level scores, teamwork climate level scores indicated a slightly lower median value for hip/knee compared to the other two procedure types (48.1 compared to 55.6 and 54.3 respectively, bottom part of Table 1).

For peer review only - http://bmjopen.bmj.com/site/about/guidelines.xhtml

Table 1: NNIS adjusted SSI rate compared to safety climate and teamwork scores, stratified by surgery type (NNIS: National Nosocomial Infections Surveillance)

N (%) / median [IQR]	**Hip and knee arthroplasty**	**Colorectal**	**Cesarean section**
Safety climate (%)	45.5 [12.5, 83.3]	48.1 [4.0, 100.0]	43.3 [0.0, 66.7]
Number of hospitals	41	28	11
Number of surgeries	20’434	8’321	4’346
NNIS adjusted infection rate	1.0% [0.0, 3.0]	16.0% [7.0, 33.0]	2.0% [1.0, 4.0]
Hospital size (beds)			
<200	27 (65.9%)	13 (46.4%)	8 (72.7%)
200-499	9 (22.0%)	9 (32.1%)	2 (18.2%)
>500	5 (12.2%)	6 (21.4%)	1 (9.1%)
Number of participants	754	493	210
participants per hospital	17 [5, 53]	12 [5, 71]	12 [6, 40]
Percentage leaders	20.0% [0.0, 50.0]	21.4 [0.0, 80.0]	18.2% [5.9%, 50.0%)
Percentage physicians (vs. nurses)	30.0% [0.0, 100.0]	34.5 [0.0, 100.0]	20.0% [0.0, 57.5]
Teamwork (%)	48.1 [4.0, 100.0]	55.6 [7.4, 88.9]	54.3 [0.0, 100.0]
Number of hospitals	44	28	12
Number of surgeries	21’247	8’321	4’824
NNIS adjusted infection rate	1.0% [0.0, 3.0]	16.0% [7.0, 31.0]	2.0% [1.0, 4.0]
Hospital size (beds)			
<200	30 (68.2%)	13 (46.4%)	9 (75.0%)
200-499	9 (20.5%)	9 (32.1%)	2 (16.7%)
>500	5 (11.4%)	6 (21.4%)	1 (8.3%)
Number of participants	861	549	239
Participants per hospital	18 [5, 56]	14 [5, 72]	14 [5, 42]
Percentage leaders	20.0% [0.0, 50.0]	21.4% [0.0, 80.0]	19.4% [5.6, 44.4]
Percentage physicians	33.3% [0.0, 100.0]	34.4% [0.0, 100.0]	22.0% [0.0, 57.4]

Otherwise, the characteristics were very similar to those for the safety climate endpoint. The stratification by hospital size did not reveal any major differences, although these comparisons certainly lacked power in some of the subcategories (figure S3). The SSI data used for the analysis was broadly comparable with that from the underlying Swissnoso population, with little discernible selection bias (table S2).

Plots of the safety endpoints against the standardized SSI rate for each of the surgery types showed generally decreasing trends, in line with our hypothesis that as safety climate and teamwork increase, the SSI rate drops. However, with the exception of the safety climate endpoint for hip and knee

For peer review only - http://bmjopen.bmj.com/site/about/guidelines.xhtml

For peer review only

surgeries (p=0.02 for slope in adjusted linear models, see table 2), these findings were not supported by formal statistical tests (figure 1). Similarly, *strength* of safety climate and teamwork climate, as measured by the standard deviation of the respective metric, was also inconclusive, although once again safety climate showed a promising trend (figure 2).

Table 2: Estimates following fitting of unadjusted and adjusted weighted linear models

A. Safety Climate

Endpoint:<br>% safety climate	**Hip and knee**<br>est., p-value		**Colorectal**<br>est., p-value		**Cesarean section**<br>est., p-value	
	univariable	multivariable	univariable	multivariable	univariable	multivariable
**SSI rate**	- , p=0.05	-, p=0.02	NS	-	NS	-
% managerial role	+, p=0.08	nE	+, p=0.01	-	+, p=0.1	-
% physician	+, p=0.08	+, p=0.03	NS	-	NS	-
Hospital size		NS		-		-
<200	reference		reference		reference	
200-499	NS		NS		NS	
500+	-, p=0.09		NS		NS	
Hospital type		-		-		-
Cantonal hospital	reference		reference		reference	
Others	NS		NS		NS	
University	NS		NS		NS	

For peer review only

For peer review only - http://bmjopen.bmj.com/site/about/guidelines.xhtml

B. Teamwork

Endpoint: % teamwork climate	**Hip and knee** est., p-value		**Colorectal** est., p-value		**Cesarean section** est., p-value	
	univariable	multivariable	univariable	multivariable	univariable	multivariable
SSI rate	NS	-	NS	-	NS	-
% managerial role	+, p=0.1	nE	+, p=0.005	-	+, p=0.1	-
% physician	+, p=0.07	+, p=0.04	+, p=0.03	-	NS	-
Hospital size				-		-
<200	reference	reference	reference		reference	
200-499	NSNS	NS	NS		NS	
500+		NS	NS		NS	
Hospital type				-		-
Kantonspital	reference	reference	reference		reference	
Others	NS	NS	NS		NS	
University	-, p=0.08	-, p=0.04	NS		NS	

est. estimate; CI confidence interval; **+** increasing trend (ie positively correlated), **-** decreasing trend (i.e., negative correlated); NS not significant at the 5% level; nE not estimated since managerial role and physician are collinear – we include "% managerial role"; hospital size and hospital type are collinear, we include hospital size

Furthermore, univariable linear models indicated that higher percentages of both managerial roles and physicians were associated with higher teamwork scores for hip/knee and colorectal surgeries. For patient-level analyses, please see table S3.

Subgroup analysis

In a subgroup analysis of hip/knee surgeries, in which a marginal association with SSI rate was apparent, we investigated whether larger hospitals are, in some sense, more critical about their own work processes, and hence have lower safety climate level scores. The right hand side of figure 3 confirms there is no difference in safety climate level scores based on hospital size (<200 beds, 200+ beds), despite a significantly higher SSI rate in larger hospitals (p=0.02). Factoring in the audit score, a measure of the SSI surveillance thoroughness but here used as a proxy for "adherence to safety-relevant protocols and standards", revealed a slightly lower median safety climate score in those with higher audit scores (i.e. more thorough adherence) for larger (200+ beds) compared to smaller hospitals (figure 4, far right set of box plots).

For peer review only

For peer review only - http://bmjopen.bmj.com/site/about/guidelines.xhtml

Missing data analysis

We multiply imputed 20 complete data sets (N=2'703 survey participants, compared to N=754 in the complete case analysis) making the missing at random assumption, including responses from all questions (safety climate and teamwork climate), along with the relevant covariates from table 1 in the imputation model, and then repeated the primary analysis for knee and hip arthroplasties. We compared descriptive statistics and the linear model estimates with those from the complete case analysis. Following multiple imputation (MI) there was an increase in physician (and "persons with managerial role") participation, indicating that physicians were less likely to complete the questionnaire (supplementary material table M1, e.g., 30% to 44% for physicians for the safety climate endpoint, p<0.001). Otherwise, the results were similar with slight, but not statistically significant, increases in safety climate and teamwork level score for the multiply imputed data (table M2).

Discussion

The idea that a good safety climate in an institution is associated with good safety outcomes for its patients is prominent in health services research. By their incidence and susceptibility to prevention measures, SSI rates are a suitable safety outcome to be linked to measurements of safety climate. As the Swiss assessment of SSI is of high quality (including infections becoming obvious after discharge from the hospital and the surveillance quality being audited on a biannual basis), this study represented a good opportunity to better understand the relationship between safety climate and safety outcomes. Indeed, this study revealed some associations between climate level and SSI rate in the expected direction, that is, the higher the safety climate, the lower the SSI rate. However, only for hip and knee surgeries was a formal statistical association observed. As implant surgery is "cleaner" than the other surgery types, it is probable that the diligence of adhering to preventive measures is better reflected in the respective infection outcomes; this may explain why the association was most salient for these surgeries. Overall, however, this study did not empirically support the expected associations between safety and teamwork climate among Swiss OR personnel and SSI rates in a strong way. This result

For peer review only - http://bmjopen.bmj.com/site/about/guidelines.xhtml

may be interpreted in two ways: that a), safety climate is not as important as other factors that have not been assessed (such as knowledge of personnel or surgical technique used) or that b) safety climate was not assessed specific and well enough to uncover existing strong associations.

Investigating the role of climate *strength* did not yield coherent and easily interpretable results, neither for safety climate nor for teamwork climate. We expected higher strength (i.e., higher agreement of climate perceptions) to be reflected in lower SSI rates. When plotting the strength scores against the infection rates, this assumption was confirmed for knee and hip surgeries as well as for cesarean sections. However, for colorectal surgeries and for the relationship between teamwork climate and cesarean section SSI rates, the direction of the association was contrary to our expectation. As we evaluated climate strengths per hospital, these mixed findings might be traced back to the frequent ad hoc formation of surgical teams which makes it hard to define team boundaries and to develop aligned perceptions of climate.

To investigate whether larger organizations are more safety-conscious and therefore have a more critical view of their own environment, we compared SSI rates and safety climate levels between larger and smaller hospitals for the arthroplastic surgery sample, but there was no difference in climate that could have corroborated this idea. In a further step, we explored whether the thoroughness of the SSI surveillance process was associated with safety climate for hip/knee surgeries (in which a marginal association with SSI rate was encountered): Larger hospitals in fact tended to have lower safety climate scores, the higher their surveillance audit scores were. Here, we are interpreting a high audit score as a marker for the ability to comply with external regulations and to follow through in implementing procedures. A hospital's workforce that is capable of maintaining a high-quality surveillance and reporting process according to national requirements may therefore be less likely to report overly optimistic safety climate scores. Such an organization obviously does much to adhere to standards and protocols, and staff may be enabled to provide a more realistic, or even critical, evaluation of teamwork and safety climate. The combination of low safety climate scores with high audit scores could therefore point to larger hospitals having a higher 'sensitivity to their operations', which is an important feature of high-reliability organizations [28].

For peer review only - http://bmjopen.bmj.com/site/about/guidelines.xhtml

Having a managerial role or being a physician had a positive influence on climate levels for two surgery types. This result is in line with earlier research indicating that persons with managing roles have a more positive view on safety climate [19,29,30], and with reports of physicians evaluating safety climate more optimistically than nurses [31], although that difference is not as clear as for managerial roles [32]. Future studies will need to shed light on the origins of differences in safety climate perceptions and their potential effects on safety outcomes.

We decided to use the safety climate scale *and* the teamwork climate scale of the SAQ [21,22], as the latter specifically covers nurse-physician collaboration in two items. Differentiating the composite safety climate dimension from the specific teamwork climate dimension revealed some nuances in the results, but overall, it did not provide particularly new insights. While Fan et al. [33] in their prior study in colon surgery investigated different climate dimensions, Lin et al. [9] argued for using composite scores, a suggestion that is supported by our results.

Limitations

Climate could only be aggregated to hospital level, due to the usual ad hoc formation of surgery teams. Respondents' identification with a different group than with their hospital was not assessed and therefore some of the real-world complexity of climate perceptions may have been lost.

Both the number of patients and hospitals included in the study provide sufficient statistical power for investigating the trends in question. However, aggregation on the hospital level (and weighting) could conceal certain trends. However, a post-hoc patient level analysis without hospital level aggregation confirmed the results from the main analysis.

For the analyses following multiple imputation of missing data, the slope estimate when regressing safety climate on the SSI rate for hip/knee arthroplasties turned out to be statistically significant (p=0.003), which was not the case for the analysis including only the complete cases. We chose to include the more conservative estimates from the complete case analysis in the results. Therefore, we take the opportunity to highlight this potential bias, and the considerable effect of including more data from physicians in the analysis.

For peer review only - http://bmjopen.bmj.com/site/about/guidelines.xhtml

The assessment of the surgical site infection rates may be biased due to underreporting of infections and thus conceal certain existing associations. As the surgical site infection rates also included an assessment of potential infections 30 days after the procedure in a telephone interview, this effect is minimized compared to studies assessing SSIs before discharge.

In adding to the inconsistent and in many cases non-significant [34,35] findings of the relationship of climate level and strength with various safety outcomes, such as medication errors [36], patient evaluations [35], length of stay [14], and healthcare-associated infections [36], this study raises two areas for future research: First, safety climate surveys may not fully assess the perceptions that are actually relevant for performing specific safety behaviors such as adhering to SSI prevention measures. As Meeks et al. [5] reported, for achieving compliance with SSI prevention measures, specific interventions were needed and having a good safety climate was “simply not enough”. Groves [37] suspected that safety climate surveys only assess the declared values in an organization, which is not necessarily what guides an individual’s behaviors. For SSI rates, this would mean integrating perceptions around performing the relevant SSI prevention measures such as administering the correct antibiotic prophylaxis in a timely fashion, for example. Applying additional qualitative approaches such as observations and interviews to learn more about the attitudes and perceptions around infection prevention measures could potentially be a fruitful avenue [38]. Secondly, to study the influence of safety climate on outcomes, it is necessary to make sure that the outcome is malleable to a large degree by safety-conscious behavior. The fact that our results are clearer for arthroplastic surgery underscores this link. Thus, outcomes should be chosen that are highly sensitive to correct infection prevention behaviors and have as few confounding influences as possible.

Conclusion

There is a growing body of research developed under the assumption that safety culture needs to be improved before or concurrently with the introduction of infection prevention measures or when a specific infection prevention measure is not showing success. However, this study adds to the inconsistent results around associations between safety climate and healthcare-associated infection

For peer review only - http://bmjopen.bmj.com/site/about/guidelines.xhtml

rates. Future studies will need to apply other safety climate assessment methods, which should be tied more closely to the actual behavior regarding infection prevention measures. Thus, using an outcome measure indicating whether prevention measures have been adhered to will be an important way to learn more about the link between safety climate and safety outcomes.

For peer review only

For peer review only - http://bmjopen.bmj.com/site/about/guidelines.xhtml

CONTRIBUTORSHIP STATEMENT

Yvonne Pfeiffer: Acquisition of data; analysis and interpretation of data; drafting of manuscript; critical revision of manuscript

Andrew Atkinson: Analysis and interpretation of data; drafting of manuscript; critical revision of manuscript

Judith Maag: Acquisition of data; analysis and interpretation of data; critical revision of manuscript

Michael A Lane: Study conception and design; critical revision of manuscript

David L. B. Schwappach: Study conception and design; analysis and interpretation of data; drafting of manuscript; critical revision of manuscript

Jonas Marschall: Study conception and design; analysis and interpretation of data; critical revision of manuscript.

COMPETING INTERESTS STATEMENT

The authors declare no competing interests.

FUNDING

This work was supported by a research grant from the Swiss National Science Foundation, Nr.: 179500 (Project "Understanding the drivers of surgical site infection: Investigating and modelling the Swissnoso surveillance data (*Watussi*)"). The funding source was not involved in study design, collection, analysis, and interpretation of data gathered nor in the writing of or decision to submit the manuscript.

For peer review only

DATA SHARING STATEMENT

Data supporting this study will be available in anonymized form for additional research under the specifications set forth by Swissnoso and the ANQ (Swiss National Association for Quality Development in Hospitals and Clinics). In principle, any researcher can submit a proposal to use Swissnoso data (including complementary data used in this study), which will be reviewed by the Swissnoso board.

ETHICS APPROVAL

The Cantonal Ethics Committee (Bern, Project ID 2019-00294) approved the study.

For peer review only

For peer review only - http://bmjopen.bmj.com/site/about/guidelines.xhtml

ACKNOWLEDGEMENTS

The authors thank all nurses and physicians who responded to the survey and to the local coordinators facilitating the survey in the hospitals. We also appreciate Maria Mancuso (Clinica Luganese Moncucco) as well as Adriana Degiorgi and Céline Corti (both Ente Ospedaliero Cantonale) for facilitating the pre-test of the Italian survey items.

The authors thank all participating centers for providing their SSI surveillance data. These data were collected in collaboration with the Swiss National Association for the Development of Quality in Hospitals and Clinics (ANQ) and the National Center for Infection Prevention (Swissnoso).

Members of the Watussi Study Group (in alphabetical order): Andrew Atkinson, PhD (Inselspital, Bern University Hospital and University of Bern), Lauro Damonti, MD (Inselspital, Bern University Hospital and University of Bern), Philipp Jent, MD (Inselspital, Bern University Hospital and University of Bern), Rüdiger Külpmann, PhD (Lucerne University of Applied Sciences and Arts), Judith Maag, MPH (Inselspital, Bern University Hospital and University of Bern), Jonas Marschall, MD (Inselspital, Bern University Hospital and University of Bern), Yvonne Pfeiffer, Dr. sc. (Swiss Patient Safety Foundation), Vanja Piezzi, MD (Inselspital, Bern University Hospital and University of Bern), Luisa Salazar, PhD (Inselspital, Bern University Hospital and University of Bern), David Schwappach, PhD (Institute of Social and Preventive Medicine (ISPM), University of Bern, Switzerland

), Benoit Sicre, PhD (Lucerne University of Applied Sciences and Arts), Bernard Surial, MD (Inselspital, Bern University Hospital and University of Bern), Nicolas Troillet, MD (Central Institute of Valais Hospitals), Andreas Widmer, MD (University Hospital Basel), Marcel Zwahlen, PhD (Institute of Social and Preventive Medicine, University of Bern).

For peer review only

References

1. Ban KA, Minei JP, Laronga C, et al. American College of Surgeons and Surgical Infection Society: Surgical Site Infection Guidelines, 2016 Update. *J Am Coll Surg*. 2017;224:59–74.

2. Guerrero MA, Anderson B, Carr G, et al. Adherence to a standardized infection n reduction bundle decreases surgical site infections after colon surgery: a retrospective cohort study on 526 patients. *Patient Saf Surg*. 2021;15:15.

3. De Bono S, Heling G, Borg MA. Organizational culture and its implications for infection prevention and control in healthcare institutions. *J Hosp Infect*. 2014;86:1–6.

4. Sakamoto F, Tomoko S, Saint S, et al. Health care-associated infection prevention in Japan: The role of safety culture. *Am J Infect Control*. 2014;42:888–893.

5. Meeks DW, Lally KP, Carrick MM, et al. Compliance with guidelines to prevent surgical site infections: As simple as 1-2-3? *Am J Surg*. 2011;201:76–83.

6. Dixon-Woods M, Leslie M, Tarrant C, et al. Explaining Matching Michigan: an ethnographic study of a patient safety program. *Implement Sci*. 2013;8:70.

7. Zohar D. Safety climate in industrial organizations: Theoretical and applied implications. *J Appl Psychol*. 1980;65:96–102.

8. Fan CJ, Pawlik TM, Daniels T, et al. Association of Safety Culture with Surgical Site Infection Outcomes. *J Am Coll Surg*. 2016;222:122–128.

9. Lin DM, Carson KA, Lubomski LH, et al. Statewide Collaborative to Reduce Surgical Site Infections : Results of the Hawaii Surgical Unit-Based Safety Program. *J Am Coll Surg*. 2018;227:189-197.e1.

10. Zohar Dov, Luria G. A Mulitlevel Model of Safety Climate: Cross-Level Relationships Between Organization an Group-Level Climates. *J Appl Psychol*. 2005;90:616–628.Atkinson A,

11. Ginsburg L, Gilin Oore D. Patient safety climate strength: a concept that requires more attention. *BMJ Qual Saf*. 2016;25:680–687.

For peer review only - http://bmjopen.bmj.com/site/about/guidelines.xhtml

12. Weaver SJ, Weeks K, Pham JC, et al. On the CUSP: Stop BSI: Evaluating the relationship between central line-associated bloodstream infection rate and patient safety climate profile. *Am J Infect Control*. 2014;42:S203–S208.

13. Mascherek AC, Schwappach DLB. Patient safety climate profiles across time: Strength and level of safety climate associated with a quality improvement program in Switzerland: A cross-sectional survey study. *PLoS ONE*. 2017;12:e0181410.

14. Tawfik DS, Thomas EJ, Vogus TJ, et al. Safety climate, safety climate strength, and length of stay in the NICU. *BMC Health Serv Res*. 2019;19:738.

15. Eisenring M-C, Troillet N, et al. Surveillance quality correlates with surgical site infection rates in knee and hip arthroplasty and colorectal surgeries: A call to action to adjust reporting of SSI rates. *Infect Control Hosp Epidemiol*. 2021;42:1451–1457.

16. Profit J, Sharek PJ, Kan P, et al. Teamwork in the NICU Setting and Its Association with Health Care – Associated Infections in Very Low-Birth-Weight Infants. *Am J Perinatol*. 2017;34:1032–1039.

17. Gambashidze N, Hammer A, Wagner A, et al. Influence of Gender, Profession, and Managerial Function on Clinicians' Perceptions of Patient Safety Culture: A Cross-National Cross-Sectional Study. *J Patient Saf*. 2021;17:e280–e287.

18. Singer SJ, Falwell A, Gaba DM, et al. Patient safety climate in US hospitals: variation by management level. *Med Care*. 2008;46:1149–1156.

19. Kristensen S, Hammer A, Bartels P, et al. Quality management and perceptions of teamwork and safety climate in European hospitals. *Int J Qual Health Care*. 2015;27:498–505.

20. Morris TP, White IR, Royston P. Tuning multiple imputation by predictive mean matching and local residual draws. *BMC Med Res Methodol*. 2014;14:75.

21. Sexton JB, Helmreich RL, Neilands TB, et al. The Safety Attitudes Questionnaire: psychometric properties, benchmarking data, and emerging research. *BMC Health Serv Res*. 2006;6:44.

22. Gehring K, Mascherek AC, Bezzola P, et al. Safety climate in Swiss hospital units : Swiss version of the Safety Climate Survey. *J Eval Clin Pract*. 2015;21:332–338.

For peer review only - http://bmjopen.bmj.com/site/about/guidelines.xhtml

23. LeBreton JM, Senter JL. Answers to 20 Questions About Interrater Reliability and Interrater Agreement. *Organ Res Methods*. 2008;11:815–852.

24. Zohar Dov, Luria G. A Mulitlevel Model of Safety Climate: Cross-Level Relationships Between Organization an Group-Level Climates. *J Appl Psychol*. 2005;90:616–628.

25. Schneider B, Salvaggio AN, Subirats M. Climate strength: a new direction for climate research. *J Appl Psychol*. 2002;87:220–229.

26. Culver DH, Horan TC, Gaynes RP, et al. Surgical wound infection rates by wound class, operative procedure, and patient risk index. *Am J Med*. 1991;91:S152–S157.

27. Kuster SP, Eisenring M-C, Sax H, et al. Structure, Process, and Outcome Quality of Surgical Site Infection Surveillance in Switzerland. *Infect Control Hosp Epidemiol*. 2017;38:1172–1181.

28. Weick KE, Sutcliffe KM, Obstfeld D. Organizing for high reliability: Processes of collective mindfulness. In: Research in Organizational Behavior, Vol. 21, 1999. 1999:81–123.

29. Gambashidze N, Hammer A, Wagner A, et al. Influence of Gender, Profession, and Managerial Function on Clinicians' Perceptions of Patient Safety Culture. *J Patient Saf*. 2019;00:1.

30. Singer SJ, Falwell A, Gaba DM, et al. Patient safety climate in US hospitals: variation by management level. *Med Care*. 2008;46:1149–1156.

31. Makary MA, Sexton JB, Freischlag JA, et al. Operating Room Teamwork among Physicians and Nurses: Teamwork in the Eye of the Beholder. *J Am Coll Surg*. 2006;202:746–752.

32. Okuyama JHH, Galvao TF, Silva MT. Healthcare Professional's Perception of Patient Safety Measured by the Hospital Survey on Patient Safety Culture: A Systematic Review and Meta-Analysis. *Sci. World J.* 2018;2018:1–11.

33. Fan CJ, Pawlik TM, Daniels T, et al. Association of Safety Culture with Surgical Site Infection Outcomes. *J Am Coll Surg*. 2016;222:122–128.

34. Lee SE, Scott LD, Dahinten VS, et al. Safety Culture, Patient Safety, and Quality of Care Outcomes: A Literature Review. *West J Nurs Res*. 2019;41:279–304.

35. DiCuccio MH. The Relationship Between Patient Safety Culture and Patient Outcomes. *J Patient Saf*. 2015;11:135–142.

For peer review only - http://bmjopen.bmj.com/site/about/guidelines.xhtml

36. Hofmann DA, Mark B. An investigation of the relationships between safety climate and medication errors as well as other nurse and patient outcomes. *Personnel Psychology*.;59:847–869.

37. Groves PS. The Relationship Between Safety Culture and Patient Outcomes: Results From Pilot Meta-Analyses. *West J Nurs Res*. 2014;36:66–83.

38. Churruca K, Ellis LA, Pomare C, et al. Dimensions of safety culture: a systematic review of quantitative, qualitative and mixed methods for assessing safety culture in hospitals. *BMJ Open*. 2021;11:43982.

For peer review only

For peer review only - http://bmjopen.bmj.com/site/about/guidelines.xhtml

Figures

Figure 1: Scores plotted against NHSN adjusted infection rate

Bubble sizes proportional to standard error of the infection rate; weighted linear model shown (red solid) with 95% confidence interval (grey shaded); LOESS smoother added (blue dashed); weights are proportional to number of participants answering the safety climate survey in the respective hospital.

A. Hip and knee arthroplasty

i. Safety climate ii. Teamwork

NHSN: National Healthcare Safety Network

B. Colorectal surgery

i. Safety climate ii. Teamwork

C. Cesarean section

i. Safety climate ii. Teamwork

For peer review only

For peer review only - http://bmjopen.bmj.com/site/about/guidelines.xhtml

Figure 2: Standard deviation of score plotted against NHSN adjusted infection rate

A. Hip and knee arthroplasty

i. Safety climate strength

ii. Teamwork climate strength

B. Colorectal surgery

i. Safety climate strength

ii. Teamwork climate strength

C. Cesarean section

i. Safety climate strength

ii. Teamwork climate strength

Figure 3: Differences in SSI rate and safety climate according to hospital size

Figure 4: Differences in safety climate ratings according to SSI surveillance quality, stratified by hospital size

For peer review only

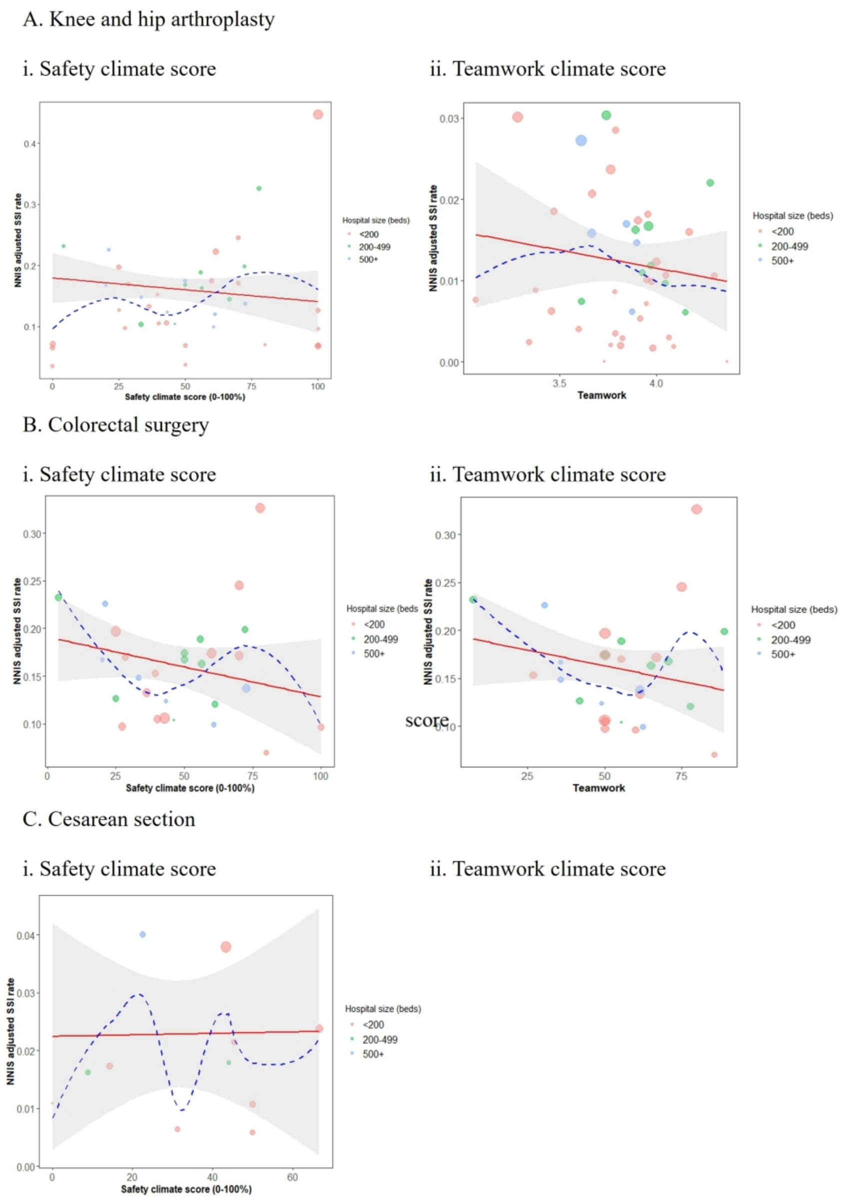

Figure 1: Scores plotted against NHSN adjusted infection rate

191x271mm (300 x 300 DPI)

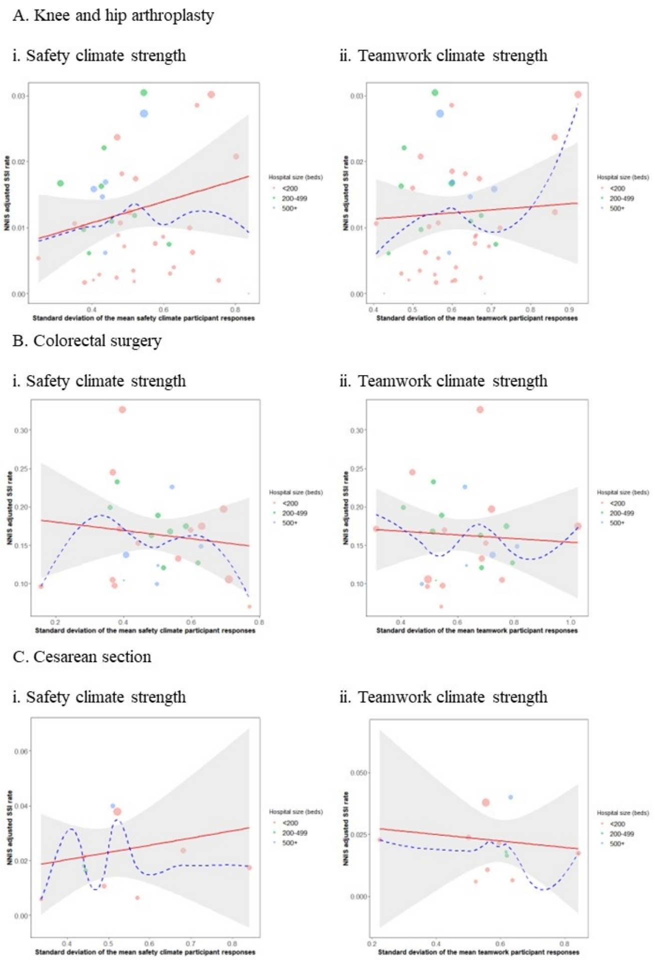

Figure 2: Standard deviation of score plotted against NHSN adjusted infection rate

190x338mm (96 x 96 DPI)

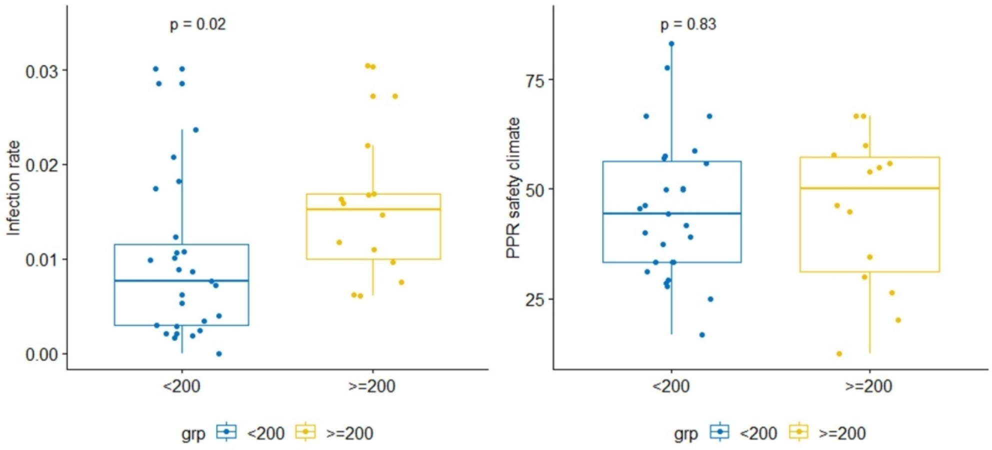

Figure 3: Differences in SSI rate and safety climate according to hospital size

250x116mm (150 x 150 DPI)

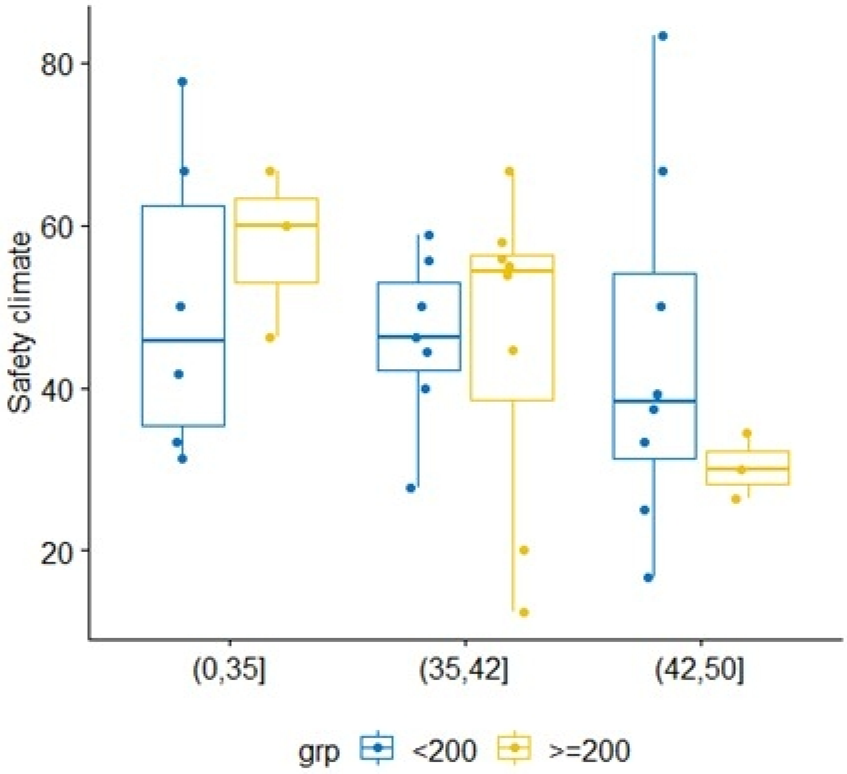

Figure 4: Differences in safety climate ratings according to SSI surveillance quality, strati-fied by hospital size

114x106mm (96 x 96 DPI)

Supplementary Tables

Table S1: Patient characteristics for all hip and knee arthroplasties, colorectal, and Cesarean section procedures in 2017-19

**Characteristic**<br>N (%) / median [IQR]	**Hip and knee**	**Colorectal**	**Cesarean section**
Number of surgeries	58’495	21’445	19’917
Number of hospitals	127	126	49
Surgical site infection	564 (1.0%)	2’973 (13.9%)	405 (2.0%)
Hospital size (beds)			
<200	42’200 (72.1%)	8’625 (40.2%)	13’991 (70.2%)
200-499	13’640 (23.3%)	8’494 (39.6%)	5’137 (25.8%)
500+	2’655 (4.5%)	4’326 (20.2%)	789 (4.0%)
Procedure Type			
Knee	24’711 (42.2%)		
Rectal		888 (4.1%)	
Class			
I (clean)	58’495 (100.0%)	0 (0.0%)	0 (0.0%)
II (potentially contaminated)	-	14’835 (69.2%)	14’281 (71.7%)
III (contaminated)	-	2’548 (11.9%)	5’235 (26.3%)
IV (infected)	-	4’062 (18.9%)	401 (2.0%)
Duration of procedure (mins)	80 [63, 102]	165 [120, 223]	40 [31, 51]
Age (years)	70 [61, 76]	69 [58, 77]	33 [30, 37]
Sex – female	32’226 (55.1%)	10’849 (50.6%)	19’917 (100.0%)
Overlong operation (*ScoreT*)	7’187 (12.3%)	9’025 (42.1%)	2’537 (12.7%)
ASA levels 3/4/5	16’777 (28.7%)	9’701 (45.2%)	1’095 (5.5%)
Minimal invasive	24’071 (41.2%)		
Endoscopic		12’261 (57.2%)	
Elective surgery	58’355 (99.8%)	15’532 (72.4%)	9’636 (48.4%)
Year			
2017	21’297 (36.4%)	6’902 (32.2%)	7’106 (35.7%)
2018	20’926 (35.8%)	7’350 (34.3%)	6’528 (32.8%)
2019	16’272 (27.8%)	7’193 (33.5%)	6’283 (31.5%)

IQR Inter Quartile Range; ASA American Society of Anesthesiology

For peer review only

Table S2: Patient characteristics of those included in the analysis compared to those from all surgeries

A. Hip and knee arthroplasty

**Characteristic**<br>N (%) / median [IQR]	**Analysis set**	**All surgeries**
Number of surgeries	20’434	58’495
Number of hospitals	41	127
Surgical site infection	205 (1.0%)	564 (1.0%)
Hospital size (beds)		
<200	15960 (78.1%)	45’475 (77.7%)
200-499	2940 (14.4%)	11’504 (18.9%)
500+	1534 (7.5%)	1’966 (3.4%)
Procedure Type		
Knee	6727 (32.9%)	24’711 (42.2%)
Class		
I (clean)	20434 (100.0%)	58’495 (100.0%)
II (potentially contaminated)	-	-
III (contaminated)	-	-
IV (infected)	-	-
Duration of procedure (mins)	76 [59, 97]	80 [63, 102]
Age (years)	70 [61, 76]	70 [61, 76]
Sex – female	10933 (53.5%)	32’226 (55.1%)
Overlong operation (*ScoreT*)	2096 (10.3%)	7’187 (12.3%)
ASA levels 3/4/5	5957 (29.2%)	16’777 (28.7%)
Minimal invasive	10123 (49.5)	24’071 (41.2%)
Elective surgery	20388 (99.8%)	58’355 (99.8%)
Year		
2017	7212 (35.3%)	21’297 (36.4%)
2018	7288 (35.7%)	20’926 (35.8%)
2019	5934 (29.0%)	16’272 (27.8%)

For peer review only

B. Colorectal procedures

**Characteristic**<br>N (%) / median [IQR]	**Analysis set**	**All surgeries**
Number of surgeries	8’321	21’445
Number of hospitals	28	126
Surgical site infection	1’301 (15.6%)	2’973 (13.9%)
Hospital size (beds)		
<200	2’442 (29.3%)	8’622 (40.2%)
200-499	3’115 (37.4%)	7’970 (37.2%)
500+	2’764 (33.2%)	4’853 (22.6%)
Procedure Type		
Rectal	396 (4.8%)	888 (4.1%)
Class		
I (clean)	0 (0.0%)	0 (0.0%)
II (potentially contaminated)	5’641 (67.8%)	14’835 (69.2%)
III (contaminated)	995 (12.0%)	2’548 (11.9%)
IV (infected)	1’685 (20.2%)	4’062 (18.9%)
Duration of procedure (mins)	155 [115, 213]	165 [120, 223]
Age (years)	69 [57, 77]	69 [58, 77]
Sex – female	4’076 (49.0%)	10’849 (50.6%)
Overlong operation (*ScoreT*)	3’131 (37.6%)	9’025 (42.1%)
ASA levels 3/4/5	3’982 (47.9%)	9’701 (45.2%)
Endoscopic	4556 (54.8%)	12’261 (57.2%)
Elective surgery	5831 (70.1%)	15’532 (72.4%)
Year		
2017	2’621 (31.5%)	6’902 (32.2%)
2018	2’799 (33.6%)	7’350 (34.3%)
2019	2’901 (34.9%)	7’193 (33.5%)

For peer review only

For peer review only - http://bmjopen.bmj.com/site/about/guidelines.xhtml

C. Cesarean sections

**Characteristic**<br>N (%) / median [IQR]	**Analysis set**	**All surgeries**
Number of surgeries	4’346	19’917
Number of hospitals	11	49
Surgical site infection	89 (2.0%)	405 (2.0%)
Hospital size (beds)		
<200	2429 (55.9%)	13’991 (70.2%)
200-499	1128 (26.0%)	5’137 (25.8%)
500+	789 (18.2%)	789 (4.0%)
Class		
I (clean)	0 (0.0%)	0 (0.0%)
II (potentially contaminated)	3313 (76.2%)	14’281 (71.7%)
III (contaminated)	993 (22.8%)	5’235 (26.3%)
IV (infected)	40 (0.9%)	401 (2.0%)
Duration of procedure (mins)	42 [33, 52]	40 [31, 51]
Age (years)	33 [30,36]	33 [30, 37]
Overlong operation (*ScoreT*)	563 (13.0%)	2’537 (12.7%)
ASA levels 3/4/5	244 (5.6%)	1’095 (5.5%)
Elective surgery	2’119 (48.8%)	9’636 (48.4%)
Year		
2017	1276 (29.4%)	7’106 (35.7%)
2018	1’520 (35.0%)	6’528 (32.8%)
2019	1550 (35.7%)	6’283 (31.5%)

For peer review only

Patient level analysis

Table S3: Uni- and multivariable estimates from the fitted logistic regression models

A. Hip and knee arthroplasty

**Endpoint:**	Univariable		Multivariable	
**SSI (0/1)**	OR (95% CI)	p-value	OR (95% CI)	p-value
Sex - female	0.8 [0.6, 1.0]	0.05	NS	
Age (in 10 year steps)		NS	-	
ASA – levels 3/4/5	2.2 [1.6, 3.0]	<0.001	2.2 [1.5, 3.0]	<0.001
Overlong operation (“scoreT”)	1.8 [1.1, 3.0]	0.02	1.8 [1.1, 2.9]	0.03
Class		nE	-	
Minimally invasive		NS		
Operation type - Knee (reference Hip)		NS		
Hospital size (number of beds)				
<200	1 (reference)			
200-500	1.5 [0.8, 2.8]	NS		
500+	2.0 [1.0, 4.0]	0.05	NS	
Safety climate (per hospital)		NS	-	
Teamwork (per hospital)		NS	-	
% managerial role (per hospital)		NS	-	
% physician (per hospital)		NS	-	

NS: not significant at the 5% level; nE: not estimated

For peer review only

For peer review only - http://bmjopen.bmj.com/site/about/guidelines.xhtml

B. Colorectal procedures

**Endpoint:**	Univariable		Multivariable	
**SSI (0/1)**	OR (95% CI)	p-value	OR (95% CI)	p-value
Sex - female	0.8 [0.7, 0.9]	0.005	0.8 [0.7, 1.0]	0.03
Age (in 10 year steps)		NS	-	
ASA – levels 3/4/5	1.7 [1.4, 2.0]	<0.001	1.3 [1.1, 1.6]	0.002
Overlong operation (“scoreT”)	1.5 [1.3, 1.8]	<0.001	1.4 [1.2, 1.7]	<0.001
Class				
II clean-contaminated	1 (reference)			
III contaminated	1.0 [0.8, 1.3]	NS	NS	
IV infected	1.7 [1.4, 2.0]	<0.001	1.3 [1.1, 1.6]	0.01
Endoscopic	0.5 [0.4, 0.6]	<0.001	0.5 [0.4, 0.6]	<0.001
Operation type - rectal	1.3 [1.0, 1.7]	0.07	1.3 [1.0, 1.7]	0.05
Hospital size (number of beds)		NS		
<200				
200-500				
500+				
Safety climate (per hospital)		NS		
Teamwork (per hospital)		NS		
% managerial role (per hospital)	1.0 [1.0, 1.0]	0.1		NS
% physician (per hospital)		NS		

For peer review only - http://bmjopen.bmj.com/site/about/guidelines.xhtml

For peer review only

B. Cesarean sections

Endpoint:	Univariable		Multivariable	
**SSI (0/1)**	OR (95% CI)	p-value	OR (95% CI)	p-value
Age (in 10 year steps)	0.7 [0.5, 1.0]	0.05		NS
ASA – levels 3/4/5		NS		
Overlong operation ("scoreT")		NS		
Class				
II clean-contaminated	1 (reference)		1 (reference)	
III contaminated	NS		NS	
IV infected	4.4 [2.3, 8.6]	0.002	3.5 [1.7, 7.2]	0.02
Hospital size (number of beds)				
<200	1 (reference)		1 (reference)	
200-500	NS		NS	
500+	2.7 [1.9, 3.9]	<0.001	2.8 [2.0, 4.0]	0.001
Safety climate (per hospital)		NS		
Teamwork (per hospital)		NS		
% managerial role (per hospital)		NS		
% physician (per hospital)	1.0 [1.0, 1.0]	0.02		NS

For peer review only - http://bmjopen.bmj.com/site/about/guidelines.xhtml

For peer review only

Table M1 Descriptive statistics of complete cases and following multiple imputation for knee and hip surgeries

	**Hip and knee**		
median [IQR]	Complete cases	Multiply imputed (K=20)	p-value
Safety climate (%)	45.5 [12.5, 83.3]	50 [16, 87]	0.09
% leaders	20% [0, 50]	25% [6, 55]	<0.001
% physician	30% [0, 100]	44% [0, 100]	<0.001
Teamwork (%)	50% [14, 89]	55 [10, 92]	0.19
% leaders	20% [0, 50]	25% [6, 55%]	<0.001
% physician	33% [0, 100]	44% [0, 100]	<0.001

For peer review only

Table M2: Comparison of estimates from the fitted weighted linear model for knee and hip surgeries

	Hip and knee slope estimate (95% CI), p-value	
Endpoint	Complete cases	Multiply imputed (K=20)
% safety climate regressed on infection rate1	-100 (-215, 15), p=0.10	-182 (-296, -68), p=0.003
% Teamwork regressed on infection rate1	-59 (-186, 67.7), p=0.37	-98 (-218, 22), p=0.11

[1]square root of the infection rate

For peer review only - http://bmjopen.bmj.com/site/about/guidelines.xhtml

Supplementary Material

Figure S1: Patient cascade and selection for hip and knee , colorectal and cesarean section procedures for the safety climate endpoint (complete case analysis).

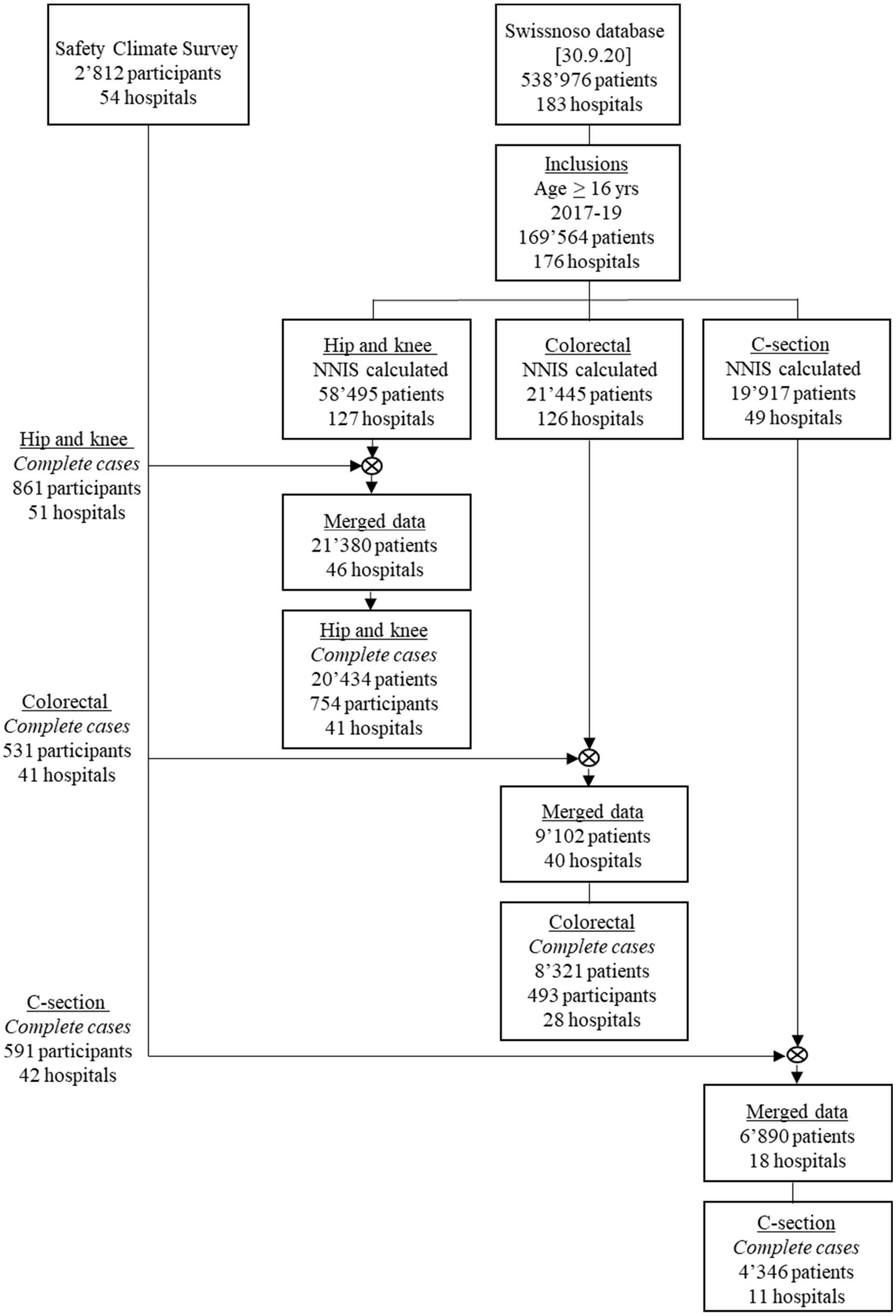

Figure S2A: Boxplot of calculated NNIS adjusted SSI rates per hospital, stratified by surgery type

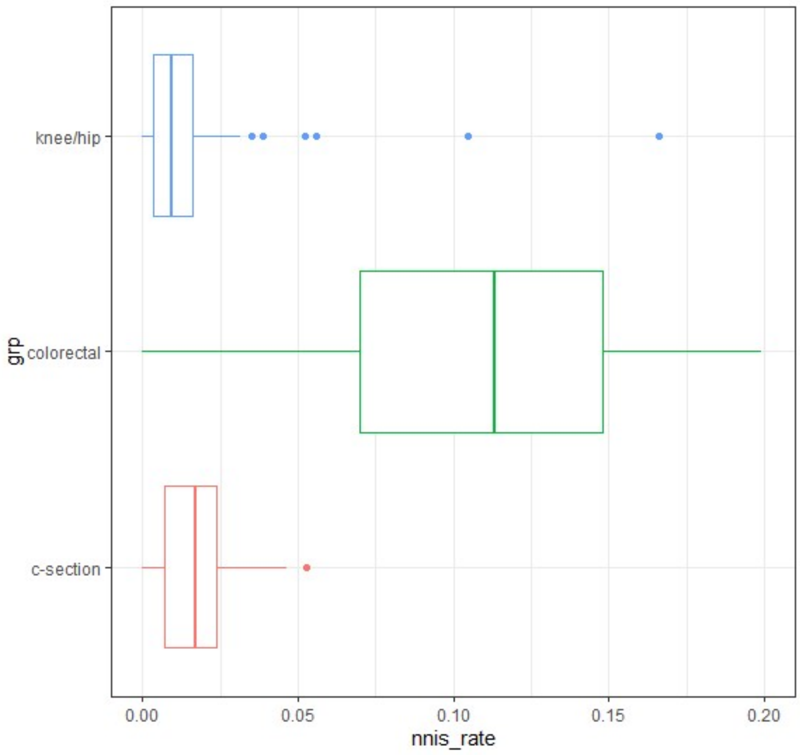

For peer review only - http://bmjopen.bmj.com/site/about/guidelines.xhtml

Figure S2B: Boxplots of calculated percentage safety climate (left) and teamwork (right) per hospital, stratified by surgery type for the complete cases from the participants

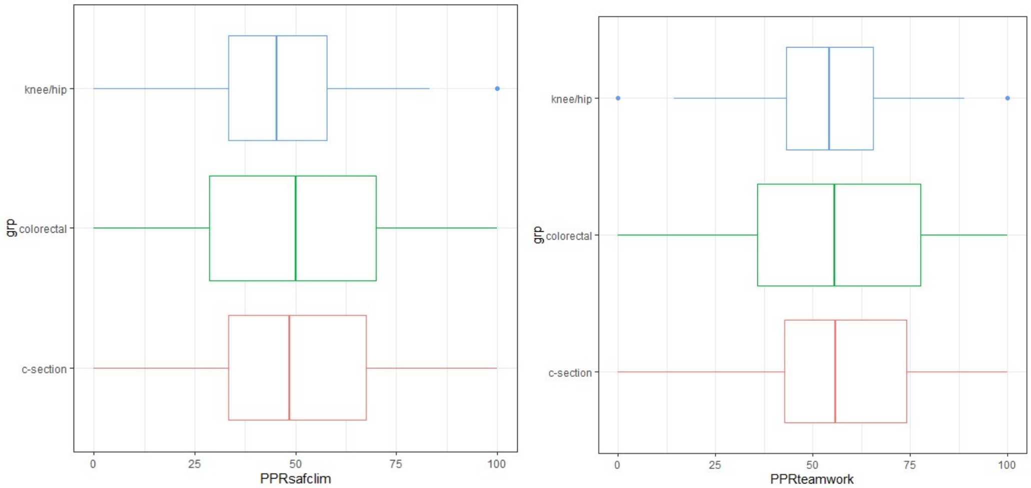

peer review only

Figure S3: Safety climate (A) and teamwork (B), stratified by hospital size

Hip and knee arthroplasty

A: Safety climate and hospital size

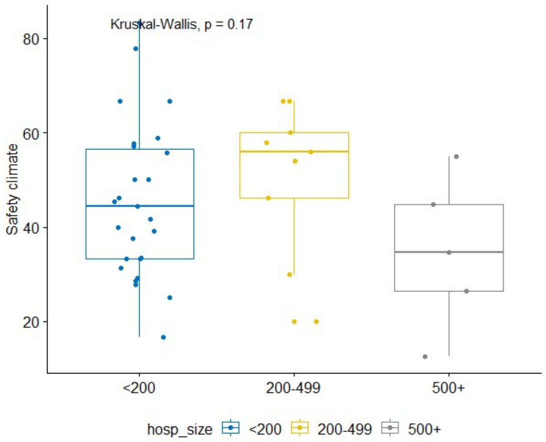

B. Teamwork and hospital size

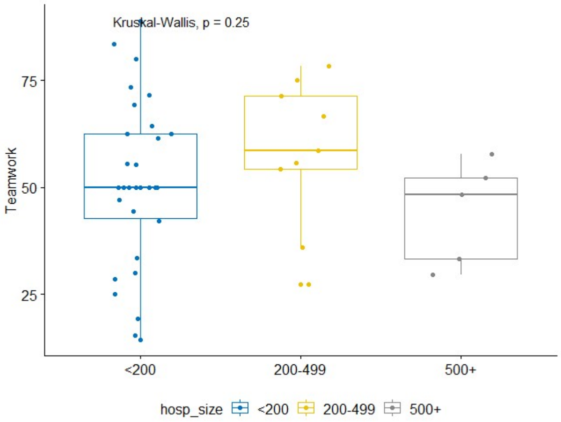

C. Infection and hospital size

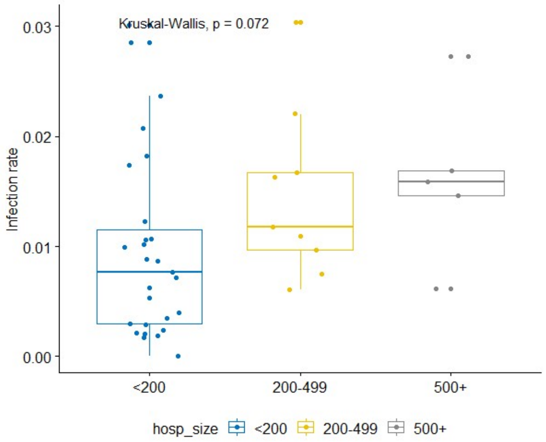

For peer review only

For peer review only - http://bmjopen.bmj.com/site/about/guidelines.xhtml

Colorectal

A: Safety climate and hospital size

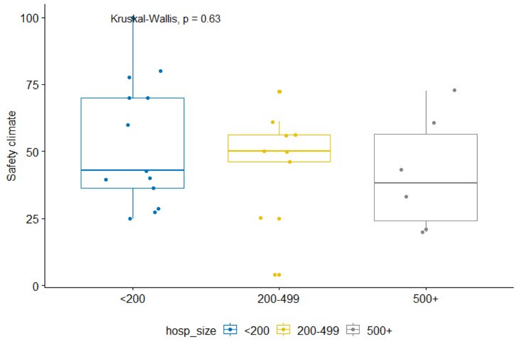

B. Teamwork and hospital size

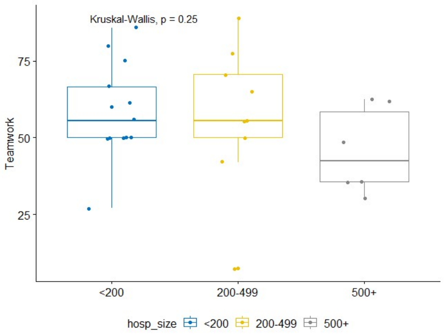

C. Infection and hospital size

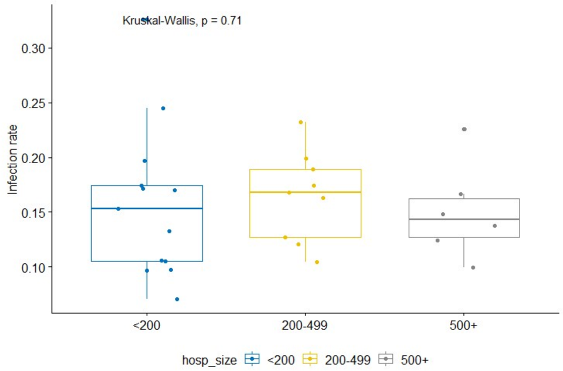

For peer review only - http://bmjopen.bmj.com/site/about/guidelines.xhtml

Cesarean section

A: Safety climate and hospital size

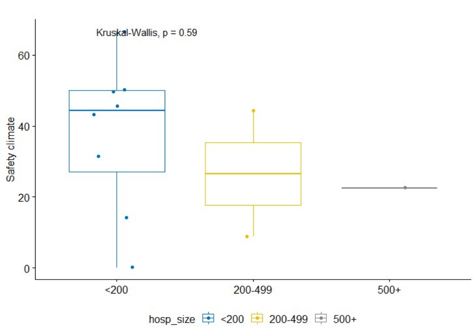

B. Teamwork and hospital size

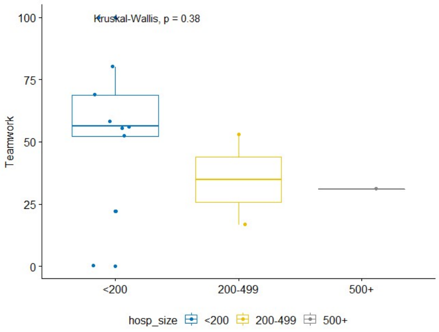

C. Infection and hospital size

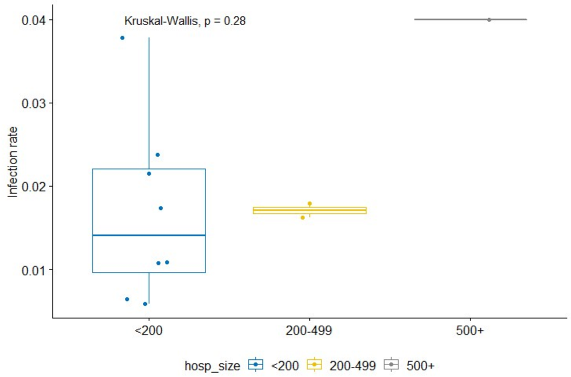

For peer review only - http://bmjopen.bmj.com/site/about/guidelines.xhtml

APPENDIX

	Recoded
**SCS Safety Climate Scale (SCS)**	
(1) The culture of this clinical area makes it easy to learn from the mistakes of others.	
(2) Medical errors** are handled appropriately in this clinical area. ** Medical error is defined as any mistake in the delivery of care, by any healthcare professional, regardless of outcome.	
(3) The senior leaders in my hospital listen to me and care about my concerns.	
(4) The physician and nurse leaders in my area listen to me and care about my concerns.	
(5) I am encouraged by my colleagues to report any safety concerns I may have.	
(6) I know the proper channels to direct questions regarding patient safety.	
(7) Leadership is driving us to be a safety- centered institution.	
(8) I receive appropriate feedback about my performance.	
(9) I would feel safe being treated here as a patient.	
(10) I am satisfied with the availability of clinical leadership (please respond to all three):	
a) Physician;	
b) Nursing;	
c) Pharmacy	
(11) Briefing personnel before the start of a shift (i.e., to plan for possible contingencies) is an important part of patient safety.	
(12) Briefings are common here.	
(13) This institution is doing more for patient safety now than it did one year ago.	
(14) I believe that most adverse events occur as a result of multiple system failures and are not attributable to one individual's actions.	
(15) The personnel in this clinical area take responsibility for patient safety.	
(16r) Management/leadership does not knowingly compromise safety concerns for productivity.	recoded
(17r) Personnel frequently disregard rules or guidelines that are established for this clinical area.	recoded
(18) Patient safety is constantly reinforced as the priority in this clinical area.	
(19r) In this clinical area, it is difficult to discuss errors.	recoded
(20) My suggestions about safety would be acted upon if I expressed them to management.	
**SCS Safety Climate Scale (SCS)**	
(1) Nurse input is well received in this clinical area.	
(2) In this clinical area, it is difficult to speak up if I perceive a problem with patient care.	recoded
(3) Disagreements in this clinical area are resolved appropriately (i.e., not who is right, but what is best for the patient).	
(4) I have the support I need from other personnel to care for patients.	
(5) It is easy for personnel here to ask questions when there is something that they do not understand.	
(6) The physicians and nurses here work together as a well-coordinated team.	

For peer review only

STROBE Statement—Checklist of items that should be included in reports of ***cross-sectional studies***

	Item No	Recommendation	Page No
**Title and abstract**	1	(*a*) Indicate the study's design with a commonly used term in the title or the abstract	1+2
		(*b*) Provide in the abstract an informative and balanced summary of what was done and what was found	2+3
**Introduction**			
Background/rationale	2	Explain the scientific background and rationale for the investigation being reported	4+5
Objectives	3	State specific objectives, including any prespecified hypotheses	5
**Methods**			
Study design	4	Present key elements of study design early in the paper	6
Setting	5	Describe the setting, locations, and relevant dates, including periods of recruitment, exposure, follow-up, and data collection	6,7,8
Participants	6	(*a*) Give the eligibility criteria, and the sources and methods of selection of participants	6,7
Variables	7	Clearly define all outcomes, exposures, predictors, potential confounders, and effect modifiers. Give diagnostic criteria, if applicable	7+8
Data sources/ measurement	8*	For each variable of interest, give sources of data and details of methods of assessment (measurement). Describe comparability of assessment methods if there is more than one group	*6,7,8*
Bias	9	Describe any efforts to address potential sources of bias	10, 15, 16
Study size	10	Explain how the study size was arrived at	11
Quantitative variables	11	Explain how quantitative variables were handled in the analyses. If applicable, describe which groupings were chosen and why	7,8,9
Statistical methods	12	(*a*) Describe all statistical methods, including those used to control for confounding	7,8, 13
		(*b*) Describe any methods used to examine subgroups and interactions	12,13
		(*c*) Explain how missing data were addressed	13
		(*d*) If applicable, describe analytical methods taking account of sampling strategy	Table M1
		(*e*) Describe any sensitivity analyses	n.a.
**Results**			
Participants	13*	(a) Report numbers of individuals at each stage of study—eg numbers potentially eligible, examined for eligibility, confirmed eligible, included in the study, completing follow-up, and analysed	10
		(b) Give reasons for non-participation at each stage	8-10
		(c) Consider use of a flow diagram	fig S1
Descriptive data	14*	(a) Give characteristics of study participants (eg demographic, clinical, social) and information on exposures and potential confounders	Tables S1, S2, S3
		(b) Indicate number of participants with missing data for each variable of interest	3
Outcome data	15*	Report numbers of outcome events or summary measures	10,11,12

For peer review only

Main results	16	(*a*) Give unadjusted estimates and, if applicable, confounder-adjusted estimates and their precision (eg, 95% confidence interval). Make clear which confounders were adjusted for and why they were included	11, 12
		(*b*) Report category boundaries when continuous variables were categorized	n.a.
		(*c*) If relevant, consider translating estimates of relative risk into absolute risk for a meaningful time period	n.a.
Other analyses	17	Report other analyses done—eg analyses of subgroups and interactions, and sensitivity analyses	12, 13
**Discussion**			
Key results	18	Summarise key results with reference to study objectives	13
Limitations	19	Discuss limitations of the study, taking into account sources of potential bias or imprecision. Discuss both direction and magnitude of any potential bias	15+16
Interpretation	20	Give a cautious overall interpretation of results considering objectives, limitations, multiplicity of analyses, results from similar studies, and other relevant evidence	13-16
Generalisability	21	Discuss the generalisability (external validity) of the study results	15-16
**Other information**			
Funding	22	Give the source of funding and the role of the funders for the present study and, if applicable, for the original study on which the present article is based	18

*Give information separately for exposed and unexposed groups.

Note: An Explanation and Elaboration article discusses each checklist item and gives methodological background and published examples of transparent reporting. The STROBE checklist is best used in conjunction with this article (freely available on the Web sites of PLoS Medicine at http://www.plosmedicine.org/, Annals of Internal Medicine at http://www.annals.org/, and Epidemiology at http://www.epidem.com/). Information on the STROBE Initiative is available at www.strobe-statement.org.

For peer review only
